# Supplementary material for: Exploring amino acid functions in a deep mutational landscape
Source: Mol Syst Biol. 2021 Jul 22;17(7):e10305. doi: 10.15252/msb.202110305 (PMC8297461; doi:10.15252/msb.202110305)
Supplement: Supplementary file 1 — Appendix [file MSB-17-e10305-s004.pdf]

Appendix

Contents

Contents 1
Appendix Figure S1 – Multi-variant Validation 2
Appendix Figure S2 – Repeat study correlation 3
Appendix Figure S3 – Studies mapped to UMAP 4
Appendix Figure S4 – Repeated positions in UMAP space 5
Appendix Figure S5 – PCA proportion of variance 6
Appendix Figure S6 – Repeat position clustering 7
Appendix Figure S7 – Relationship between number of positions and number of subtypes .. 8
Appendix Figures S8-S28 – Shared legend 9
Appendix Figure S8 - Alanine Subtype Characterisation 10
Appendix Figure S9 – Cysteine Subtype Characterisation 11
Appendix Figure S10 – Aspartate Subtype Characterisation 12
Appendix Figure S11 – Glutamate Subtype Characterisation 13
Appendix Figure S12 – Phenylalanine Subtype Characterisation 14
Appendix Figure S13 – Glycine Subtype Characterisation 15
Appendix Figure S14 – Histidine Subtype Characterisation 16
Appendix Figure S15 – Isoleucine Subtype Characterisation 17
Appendix Figure S16 – Lycine Subtype Characterisation 18
Appendix Figure S17 – Leucine Subtype Characterisation 19
Appendix Figure S18 – Methionine Subtype Characterisation 20
Appendix Figure S19 – Asparagine Subtype Characterisation 21
Appendix Figure S20 – Proline Subtype Characterisation 22
Appendix Figure S21 – Glutamine Subtype Characterisation 23
Appendix Figure S22 – Arginine Subtype Characterisation 24
Appendix Figure S23 – Serine Subtype Characterisation 25
Appendix Figure S24 – Threonine Subtype Characterisation 26
Appendix Figure S25 – Valine Subtype Characterisation 27
Appendix Figure S26 – Tryptophan Subtype Characterisation 28
Appendix Figure S27 – Tyrosine Subtype Characterisation 29
Appendix Figure S28 – SIFT4G Mutational Landscape 30
Appendix Figure S29 – Comparison of cosine distance distribution for ER and SIFT4G based subtypes 31

Appendix Figure S1 – Multi-variant Validation

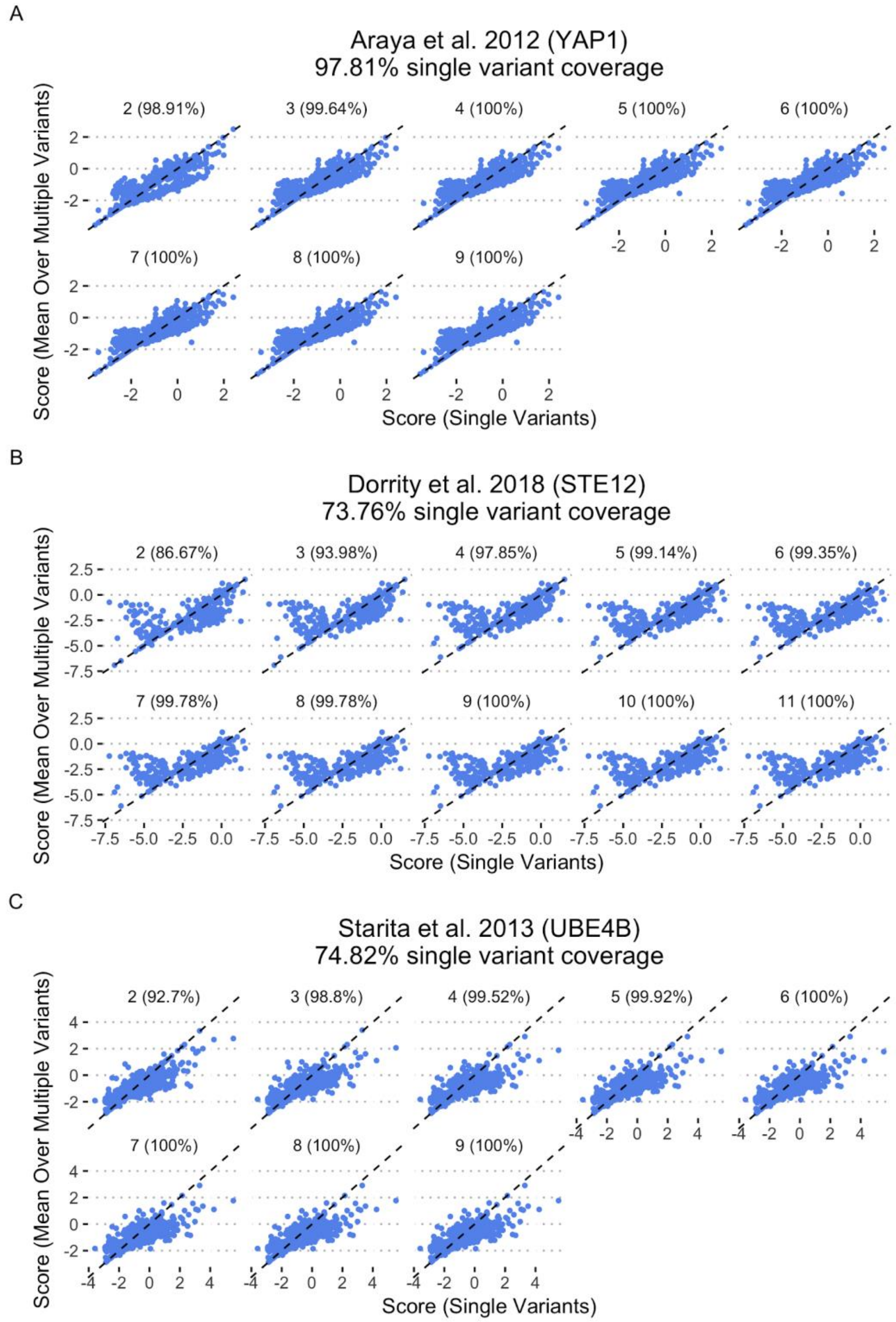

Appendix Figure S1 - Validating averaging scores from multiply mutated sequences to estimate individual substitution scores in data from Araya et al. 2012 (**A**), Dorrity et al. 2018 (**B**) and Starita et al. 2013 (**C**). Each panel plots the score for explicitly measured single variants against the average score for multi-mutants containing that variant, using sequences with at most an increasing number of variants across subpanels. Each panel and subpanel also notes the score coverage of substitutions in the data when averaging sequences with that many mutations.

Appendix Figure S2 – Repeat study correlation

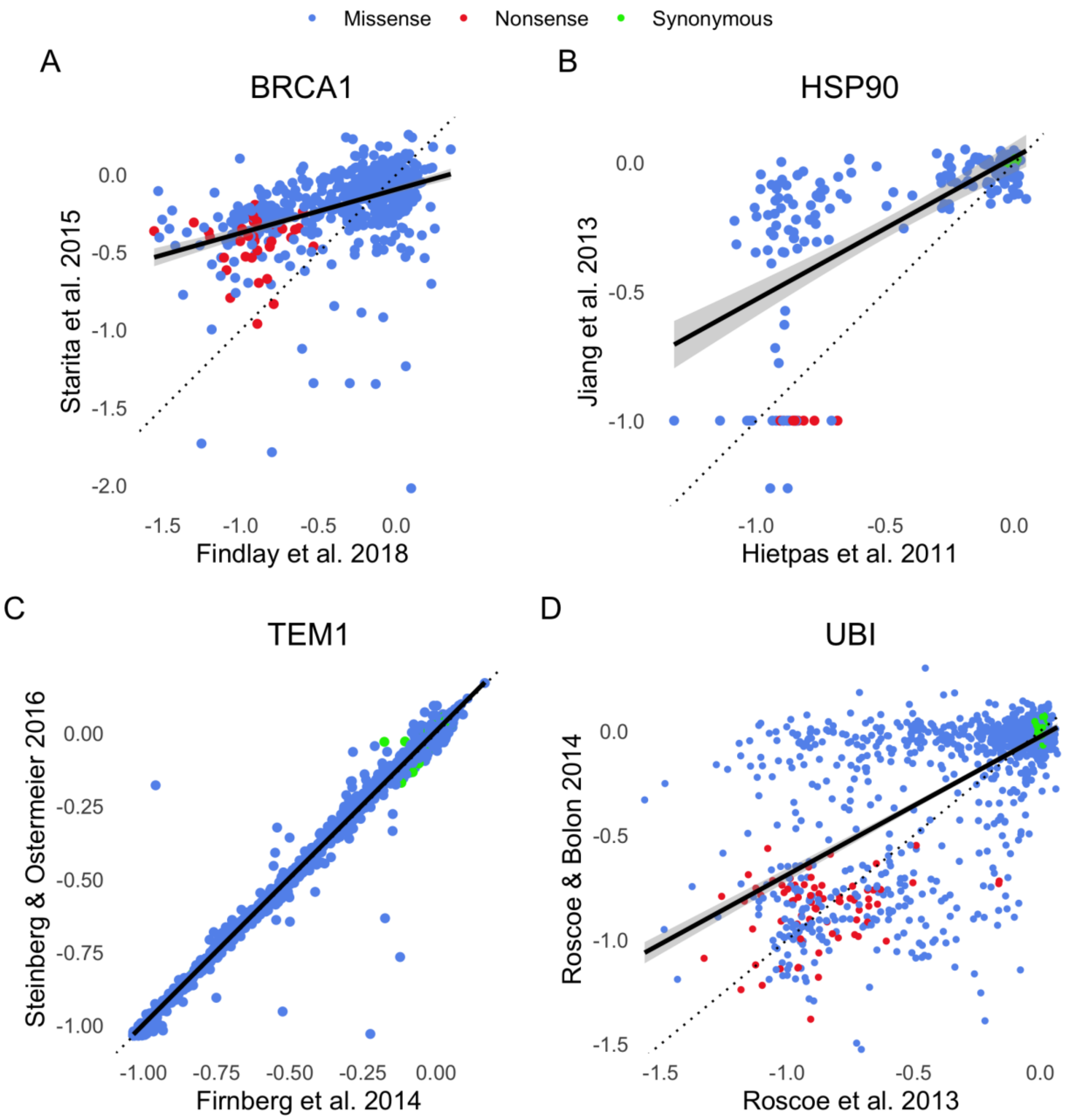

Appendix Figure S2 - Correlation between scores from studies covering the same gene. **A**: BRCA1 (from the Fields and Shendure labs) **B**: HSP9 (both studies from the Bolon lab) **C**: TEM1 (both studies from the Ostermeier lab) **D**: Ubiquitin (both studies from the Bolon lab)

Appendix Figure S3 – Studies mapped to UMAP

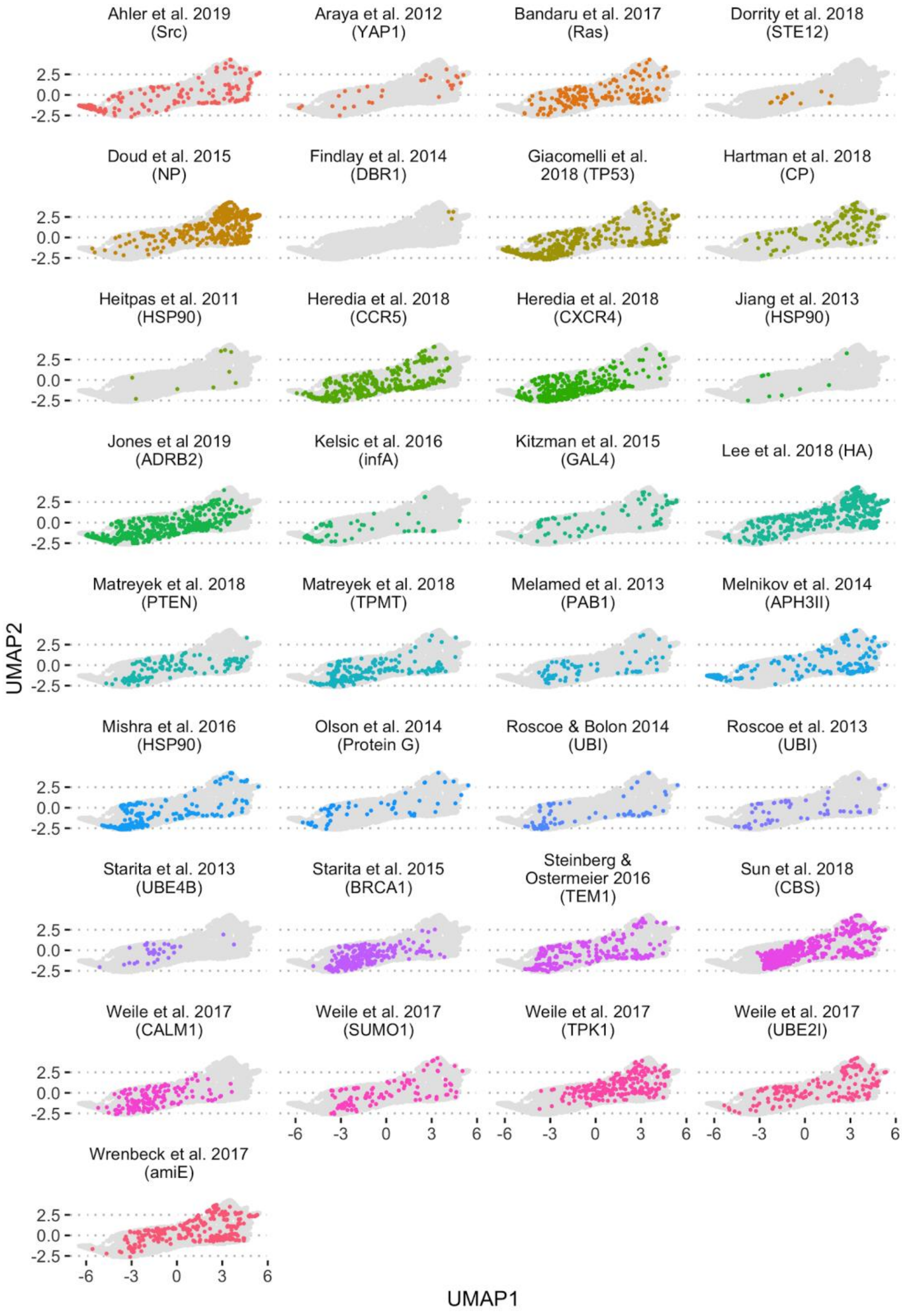

Appendix Figure S3 - Positions from each study mapped to UMAP space. The positions from each study are coloured while the overall extent of the space is shown in grey, demonstrating points span the space rather than clustering by study. The few cases where some clustering appears are in studies with very few results, which are restricted to specifically chosen functional regions and so would be expected to have atypical landscape distributions.

Appendix Figure S4 – Repeated positions in UMAP space

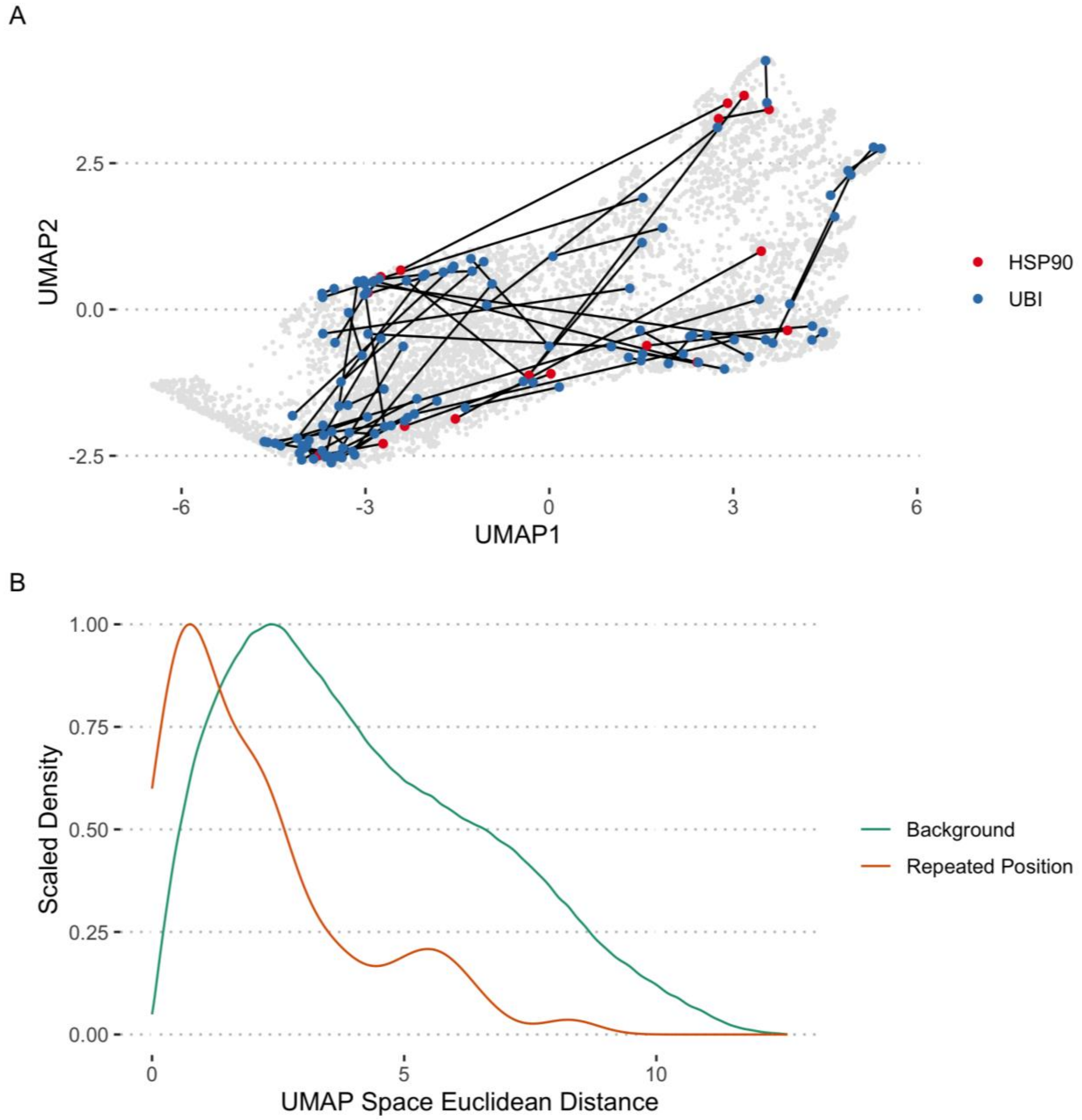

Appendix Figure S4 - Distance between repeated positions in UMAP space. **A**: Repeated positions mapped into UMAP space, with the two copies of each position linked by a line. **B**: Distribution of distances between points in UMAP space, comparing repeated positions to the background distribution of random position pairs. Repeated positions are significantly closer on average (One tailed Mann-Whitney U-Test: $p = 1.141 \times 10^{-13}$).

Appendix Figure S5 – PCA proportion of variance

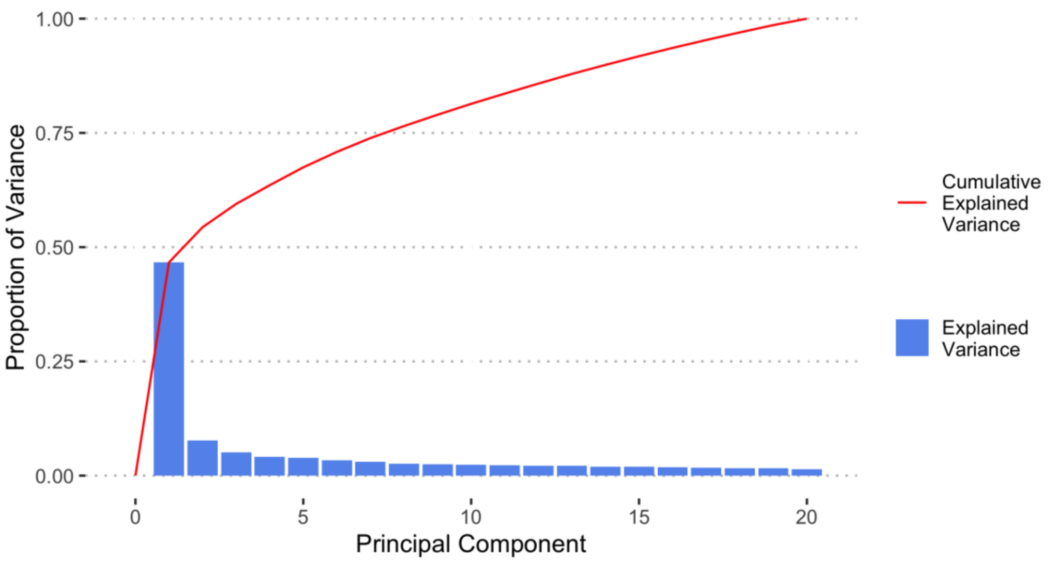

Appendix Figure S5 - Proportion of variance explained by each principal component (blue bars) and the cumulative proportion explained (red line)

Appendix Figure S6 – Repeat position clustering

A

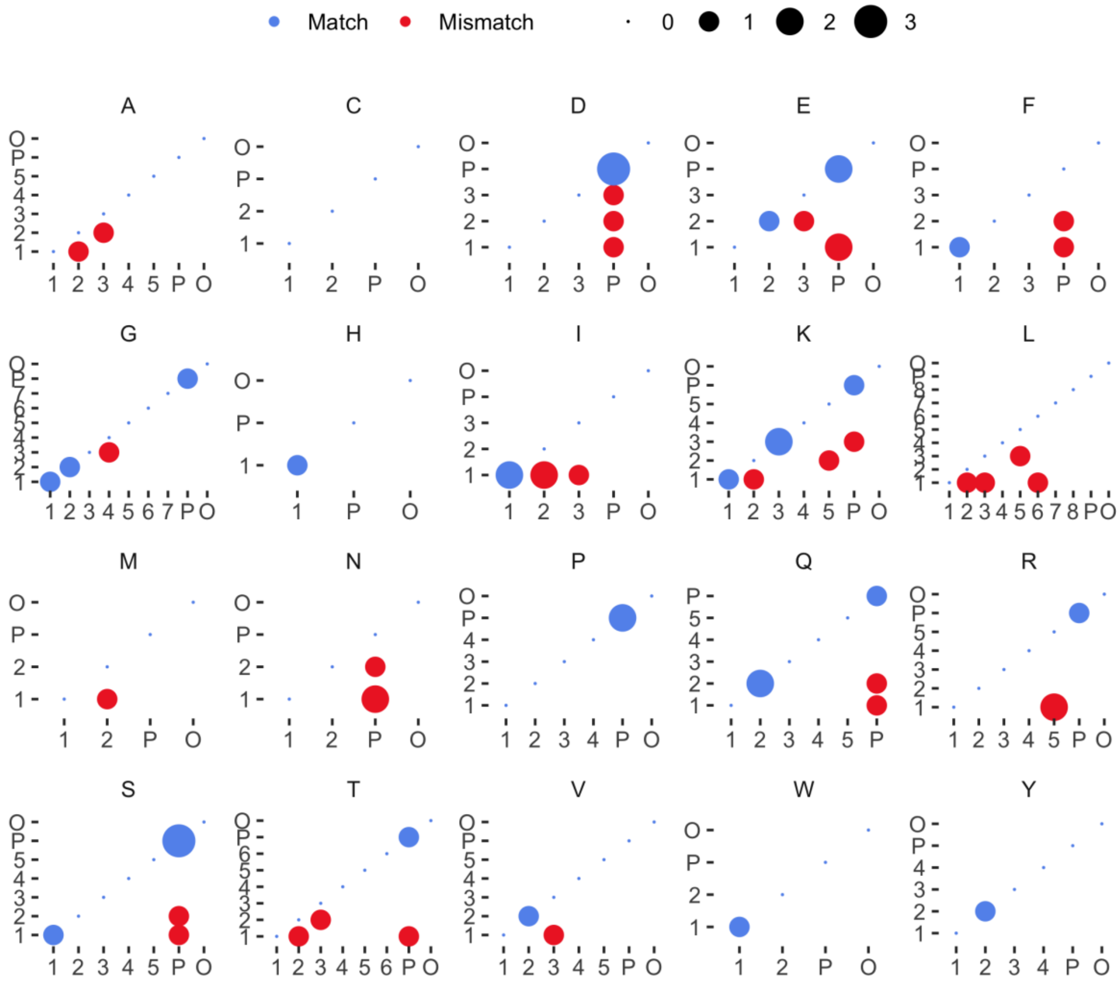

B

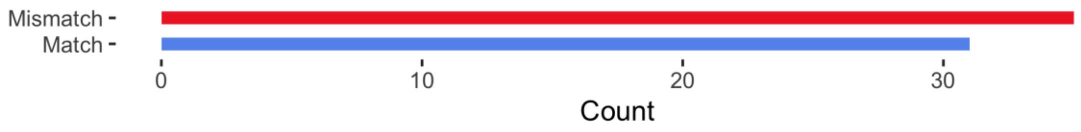

Appendix Figure S6 - **A**: Pairs of subtypes the position covered by multiple studies were assigned to, showing matches in blue and mismatches in red. Subtype number pairs are sorted such that they are always placed in the lower triangle. **B**: Count of matches and mismatches.

Appendix Figure S7 – Relationship between number of positions and number of subtypes

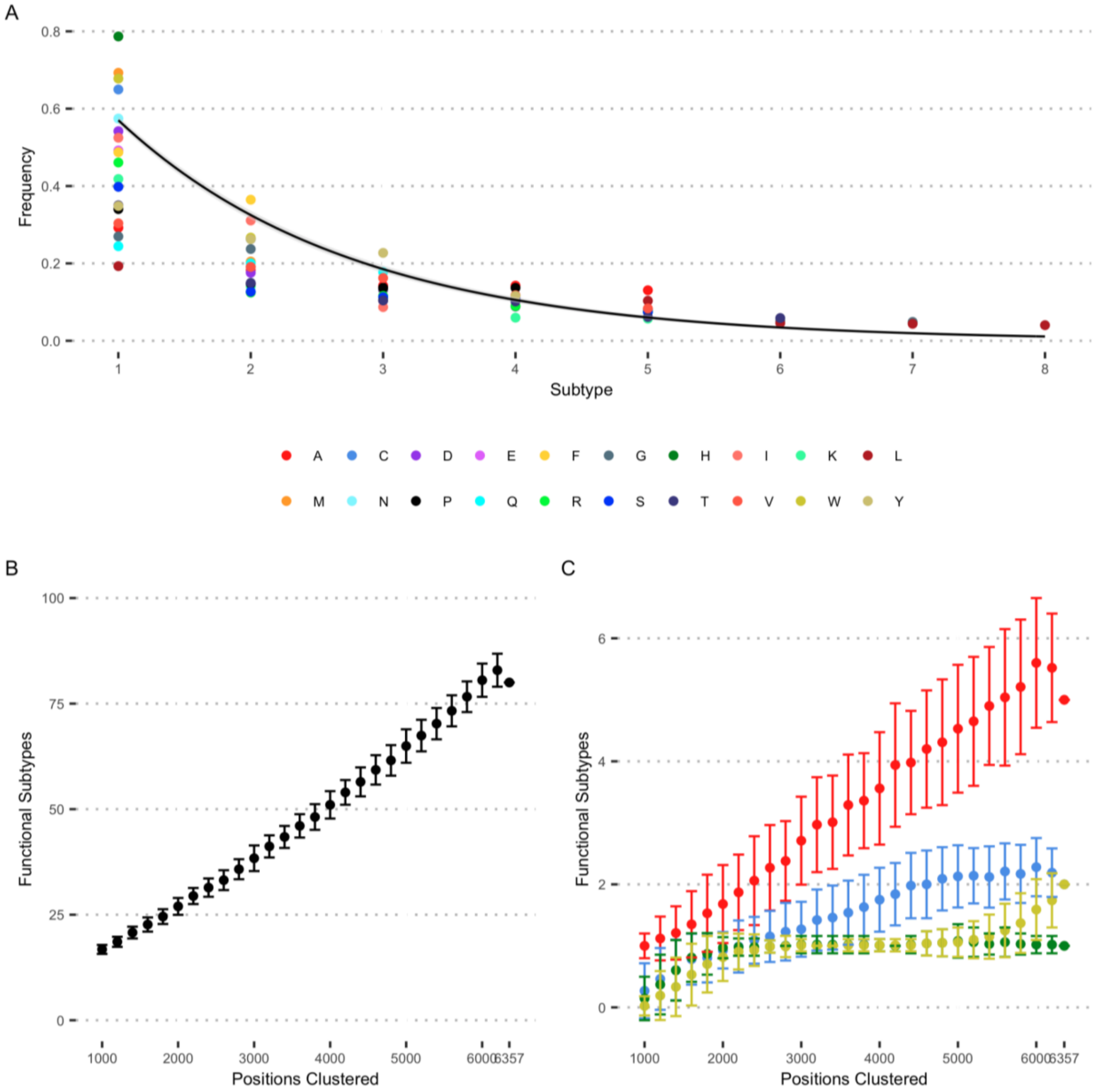

Appendix Figure S7 - **A**: Frequency of each subtype number fit by an exponential function, across all amino acids. $y = e^{-(0.56197 \pm 0.01655)x}$, $r^2 = 0.9359$, $p < 2.2 \times 10^{-16}$. **B**: Total number of subtypes identified when reclustering with an increasing number of positions from the dataset. The clustering algorithm was applied to increasingly large data subsets (increments of 200, starting at 1000) for 100 shuffles of the data. The mean and standard deviation of the number of functional subtypes (not permissive or outliers) identified at each position count from these samples is shown. **C**: Number of subtypes identified for alanine, cysteine, histidine and tryptophan, illustrating the saturation that occurs for some amino acids but not others.

Appendix Figures S8-S28 – Shared legend

Appendix Figure S8-27 - Plots characterising the subtypes of each amino acid, each following the same format. These are displayed landscape below and attached in an additional file. **A**: Number of positions assigned to each subtype. **B**: Relative probability of each subtype occurring in each secondary structure **C**: ER profiles **D**: $\log_{10}$SIFT4G score profiles **E**: Surface accessibility distributions **F**: Normalised mean profiles of each FoldX energy term for substitutions at positions of each subtype **G**: Normalised mean chemical environment profiles for positions of each subtype. These comprised the count of each type of amino acid within 10Å of the target position.

Appendix Figure S8 - Alanine Subtype Characterisation

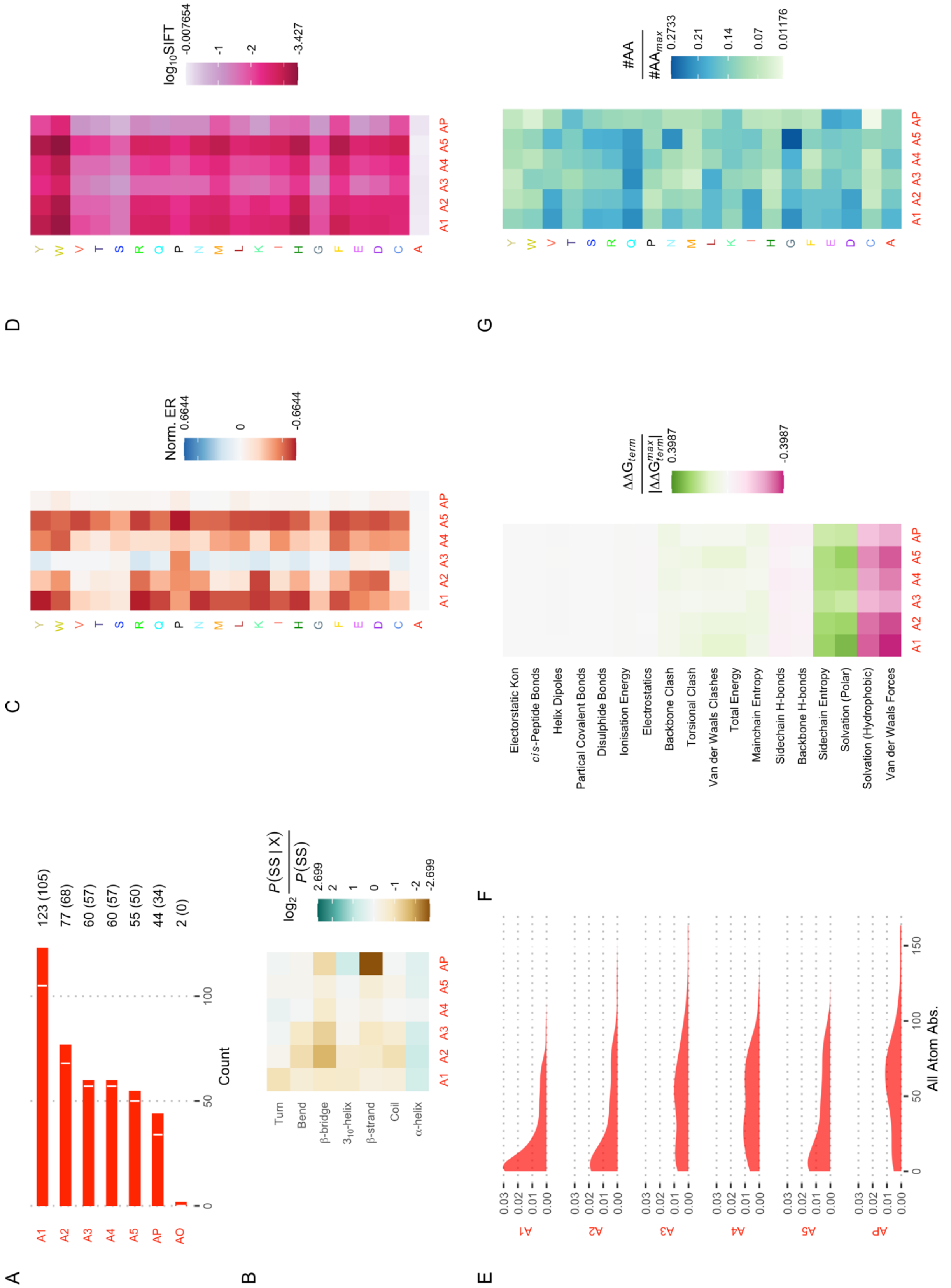

Figure S8 – Alanine Subtype Characterisation

Appendix Figure S9 – Cysteine Subtype Characterisation

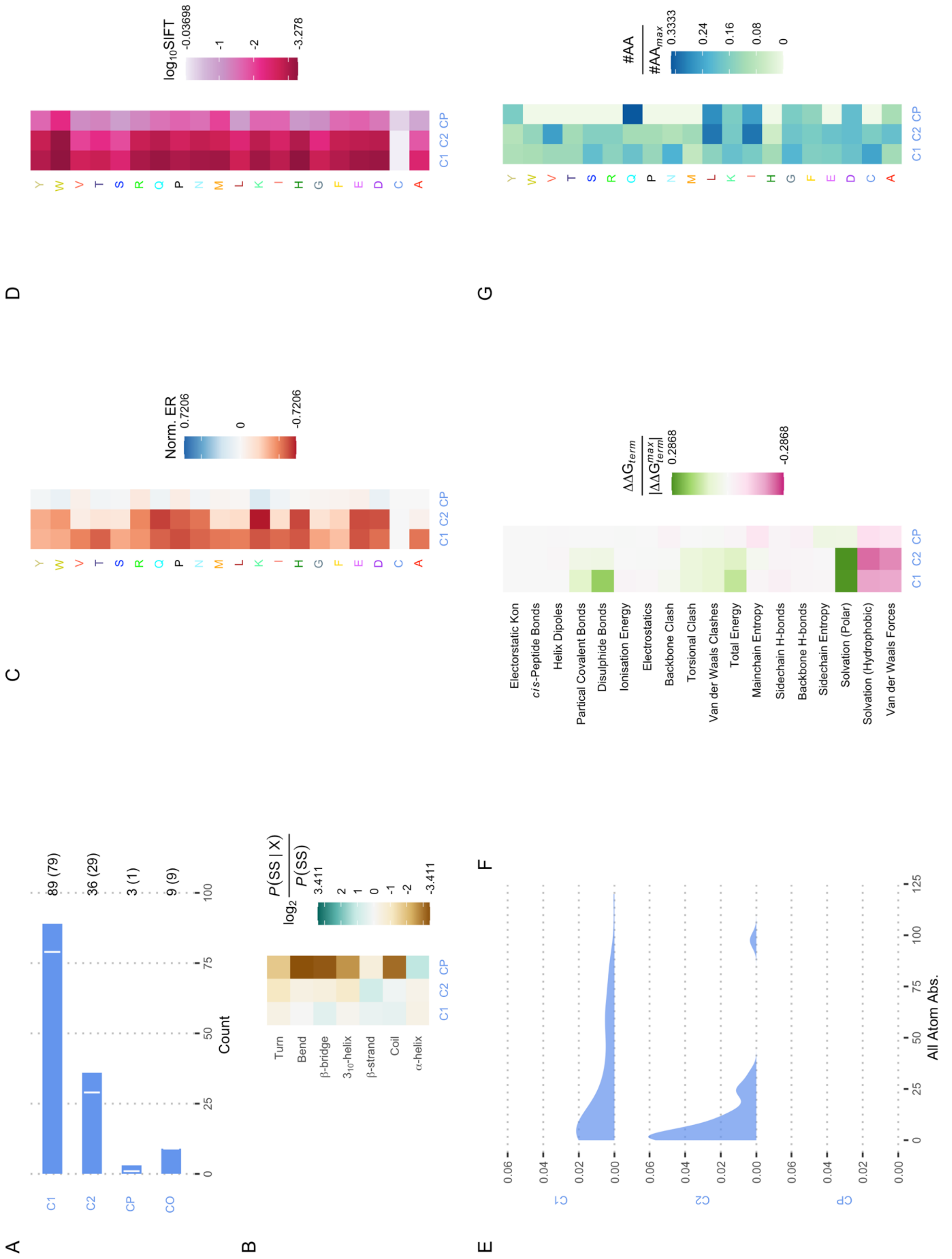

Figure S9 – Cysteine Subtype Characterisation

Appendix Figure S10 – Aspartate Subtype Characterisation

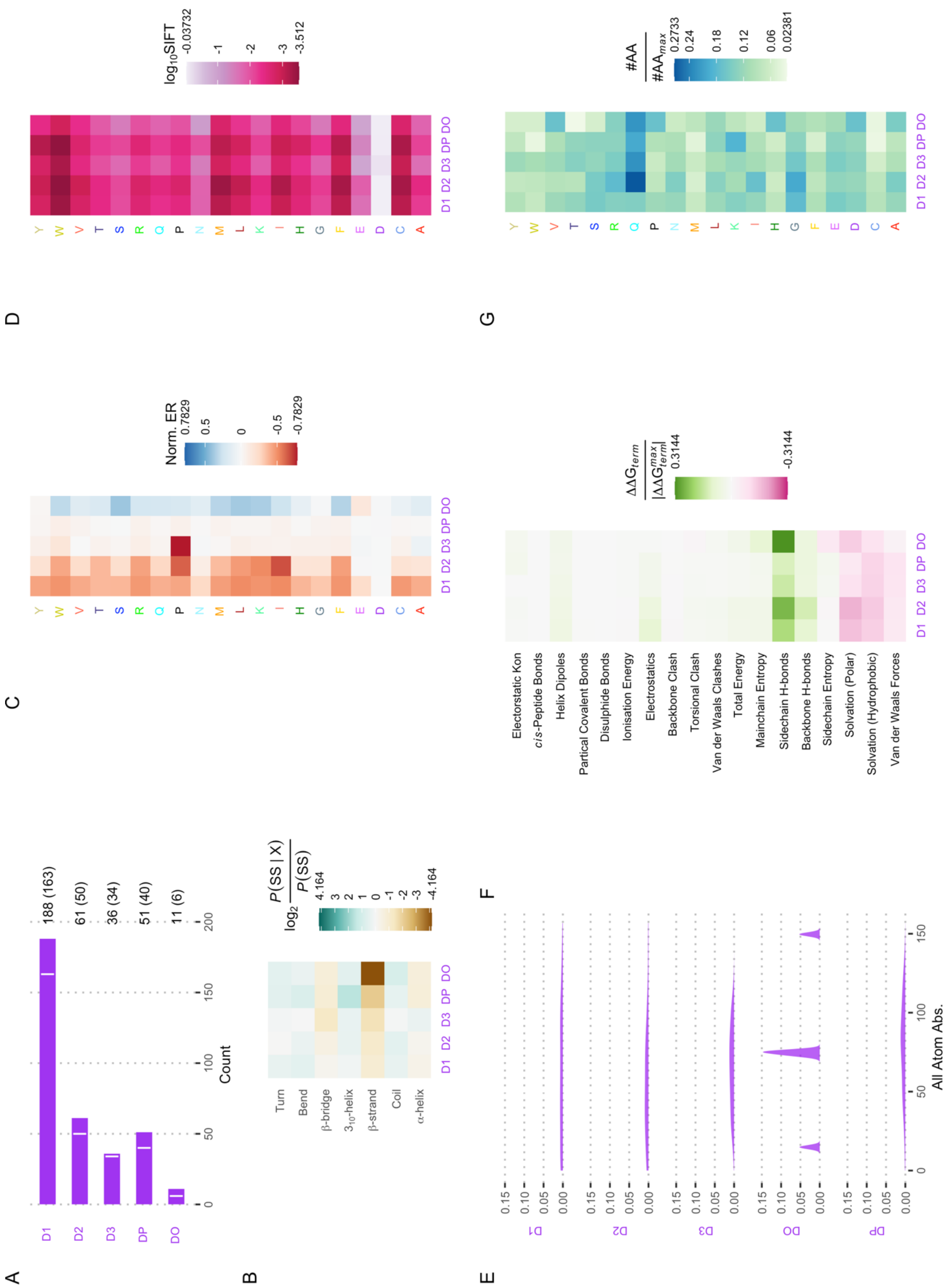

Figure S10 – Aspartate Subtype Characterisation

Appendix Figure S11 – Glutamate Subtype Characterisation

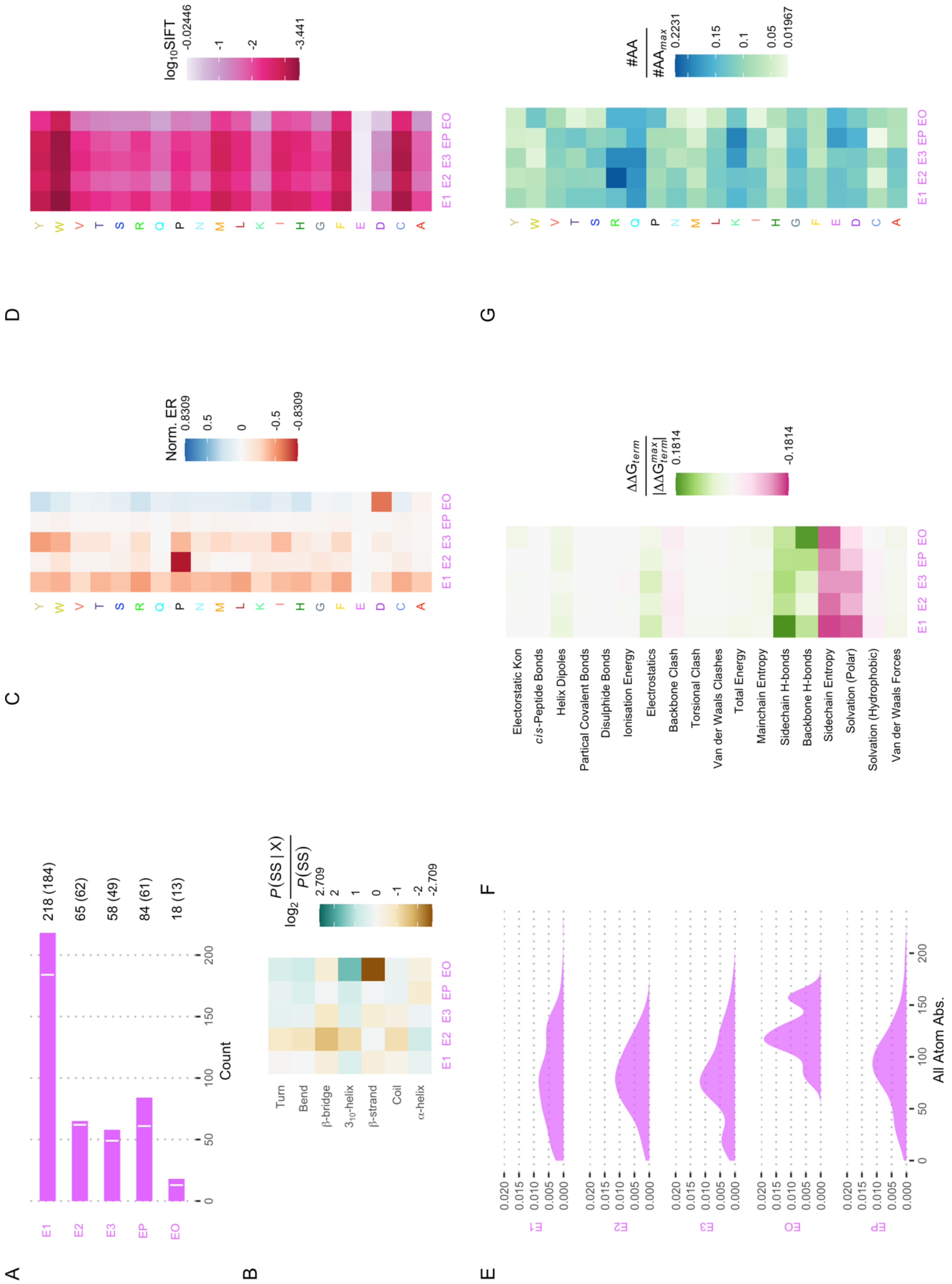

Figure S11 – Glutamate Subtype Characterisation

Appendix Figure S12 – Phenylalanine Subtype Characterisation

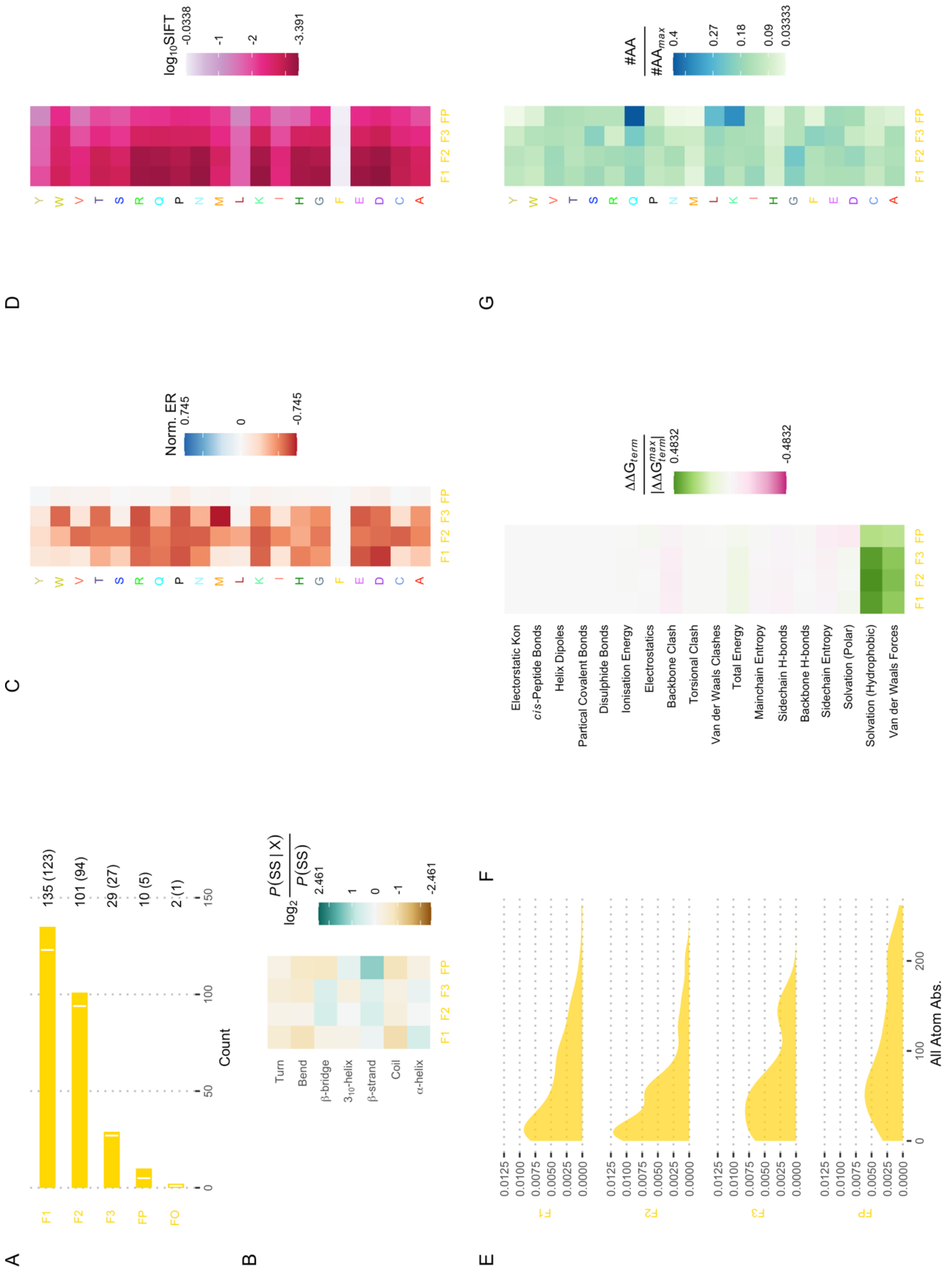

Figure S12 – Phenylalanine Subtype Characterisation

Appendix Figure S13 – Glycine Subtype Characterisation

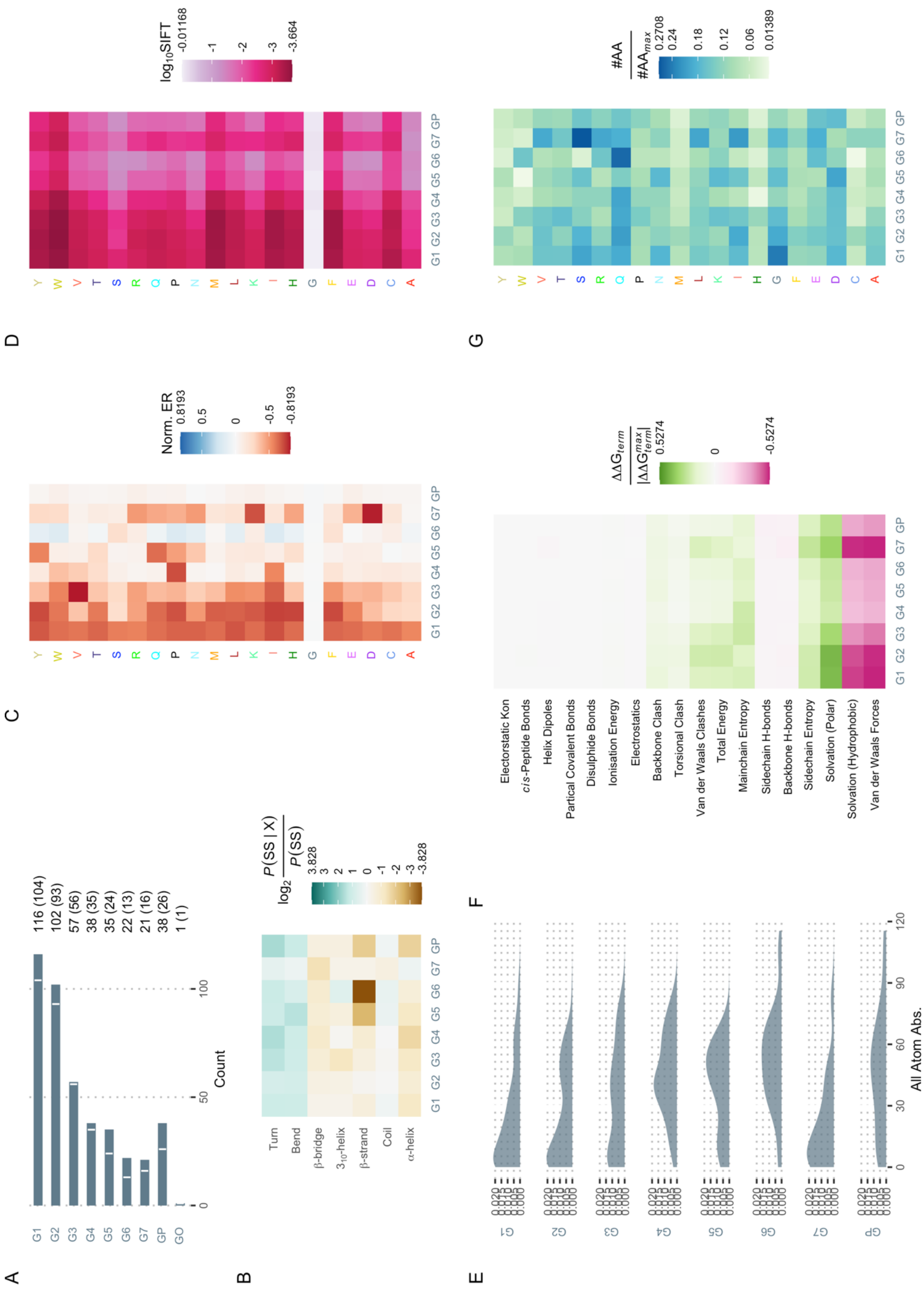

Figure S13 – Glycine Subtype Characterisation

Appendix Figure S14 – Histidine Subtype Characterisation

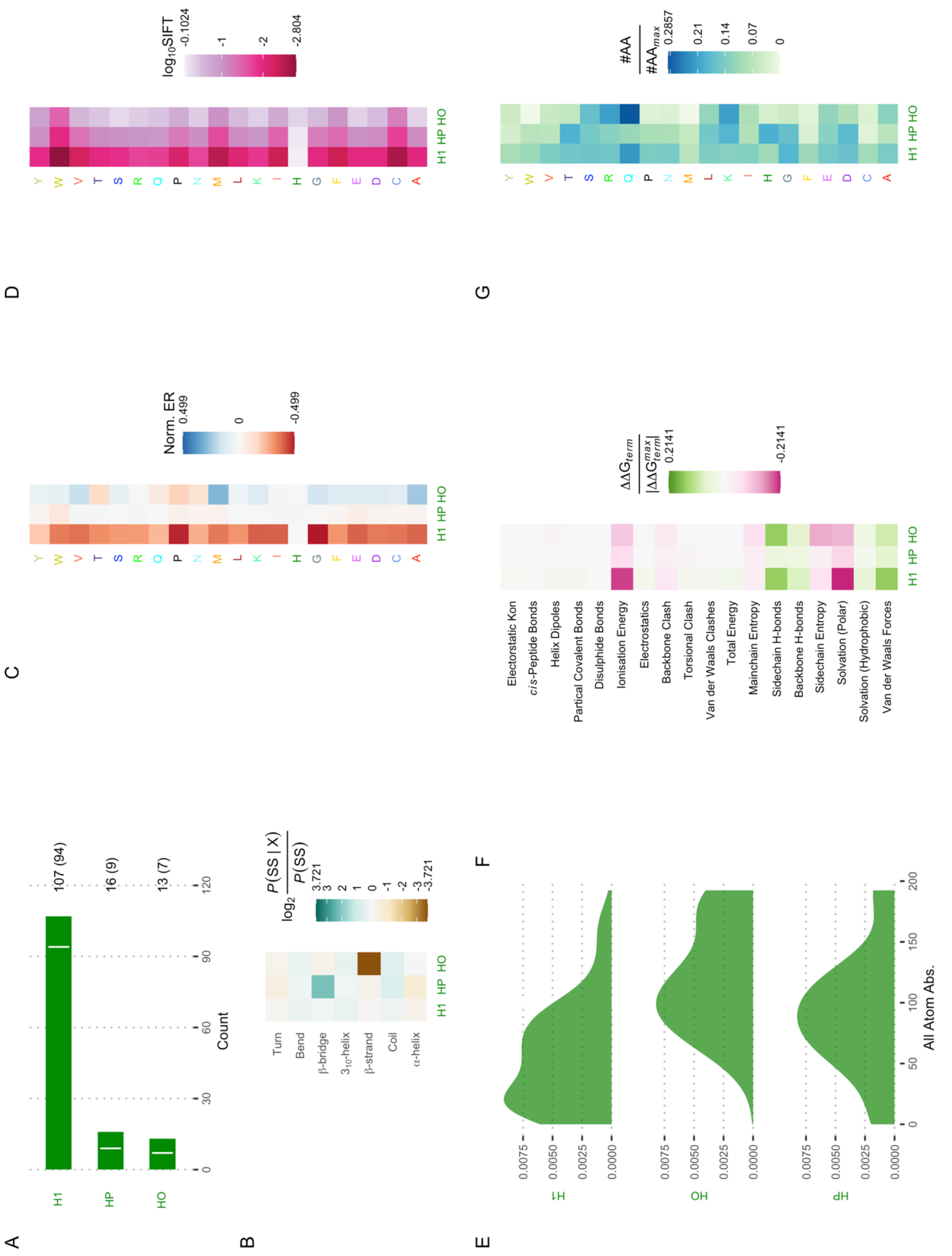

Figure S14 – Histidine Subtype Characterisation

Appendix Figure S15 – Isoleucine Subtype Characterisation

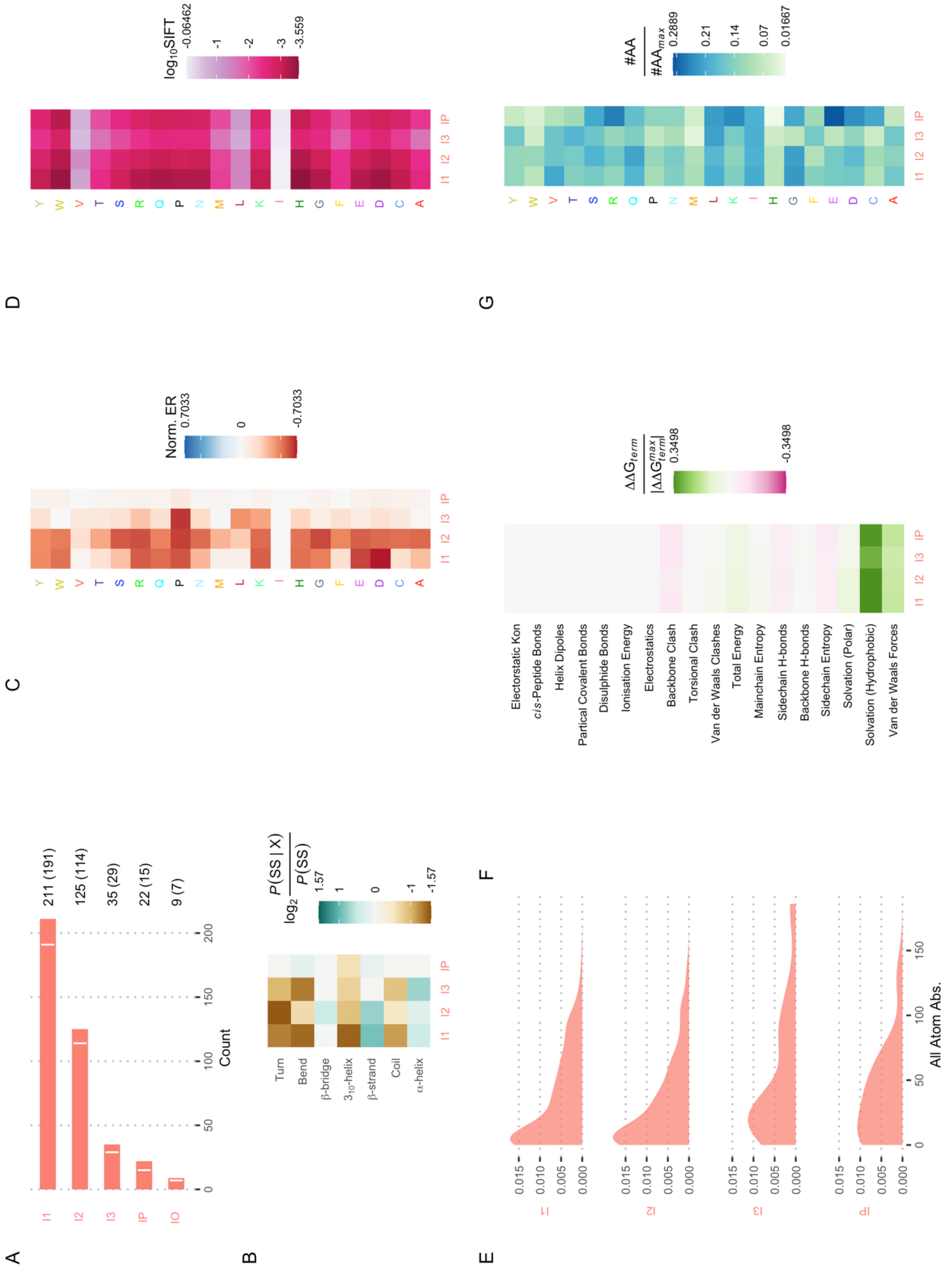

Figure S15 – Isoleucine Subtype Characterisation

Appendix Figure S16 – Lycine Subtype Characterisation

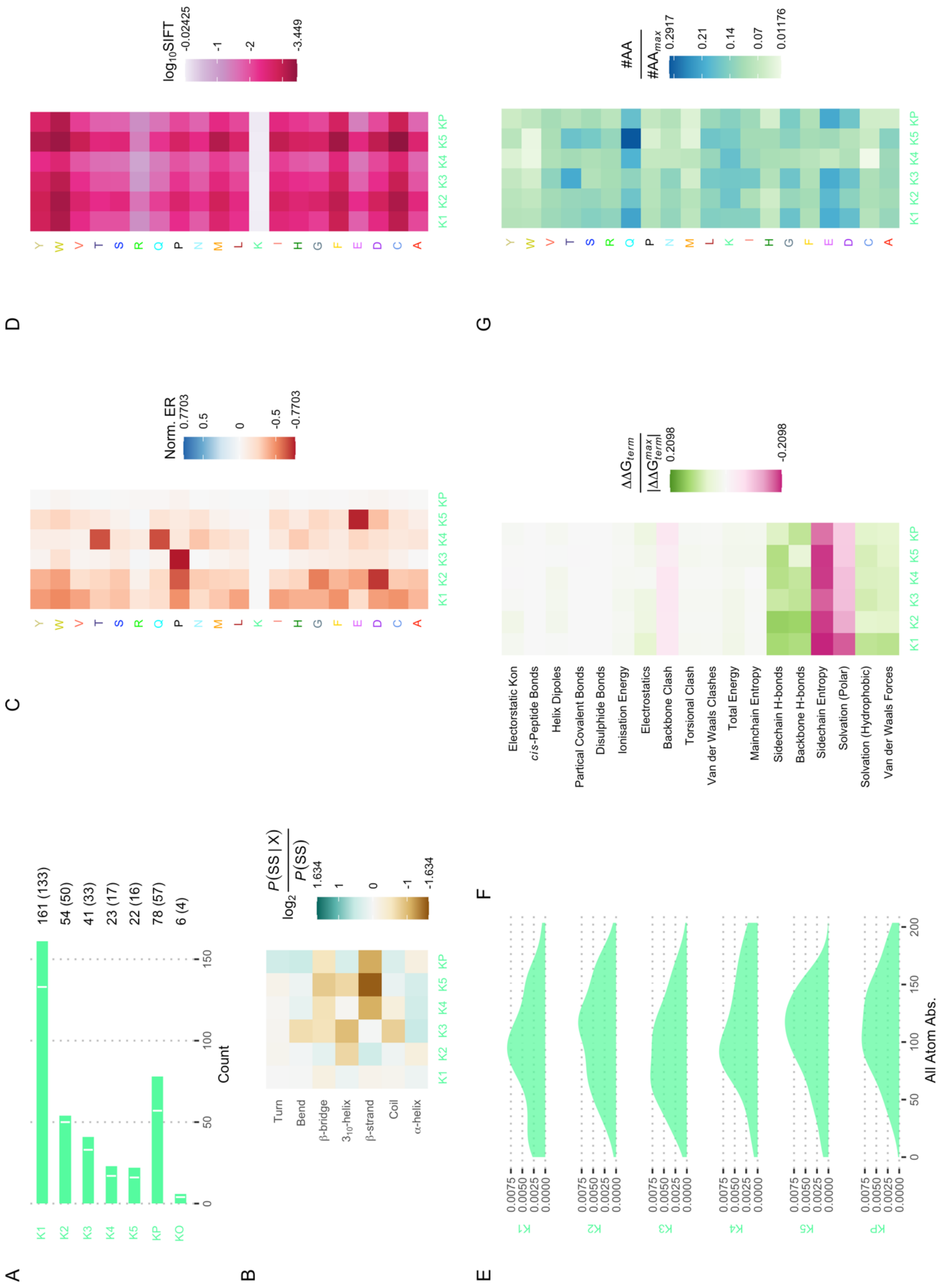

Figure S16 – Lycine Subtype Characterisation

Appendix Figure S17 – Leucine Subtype Characterisation

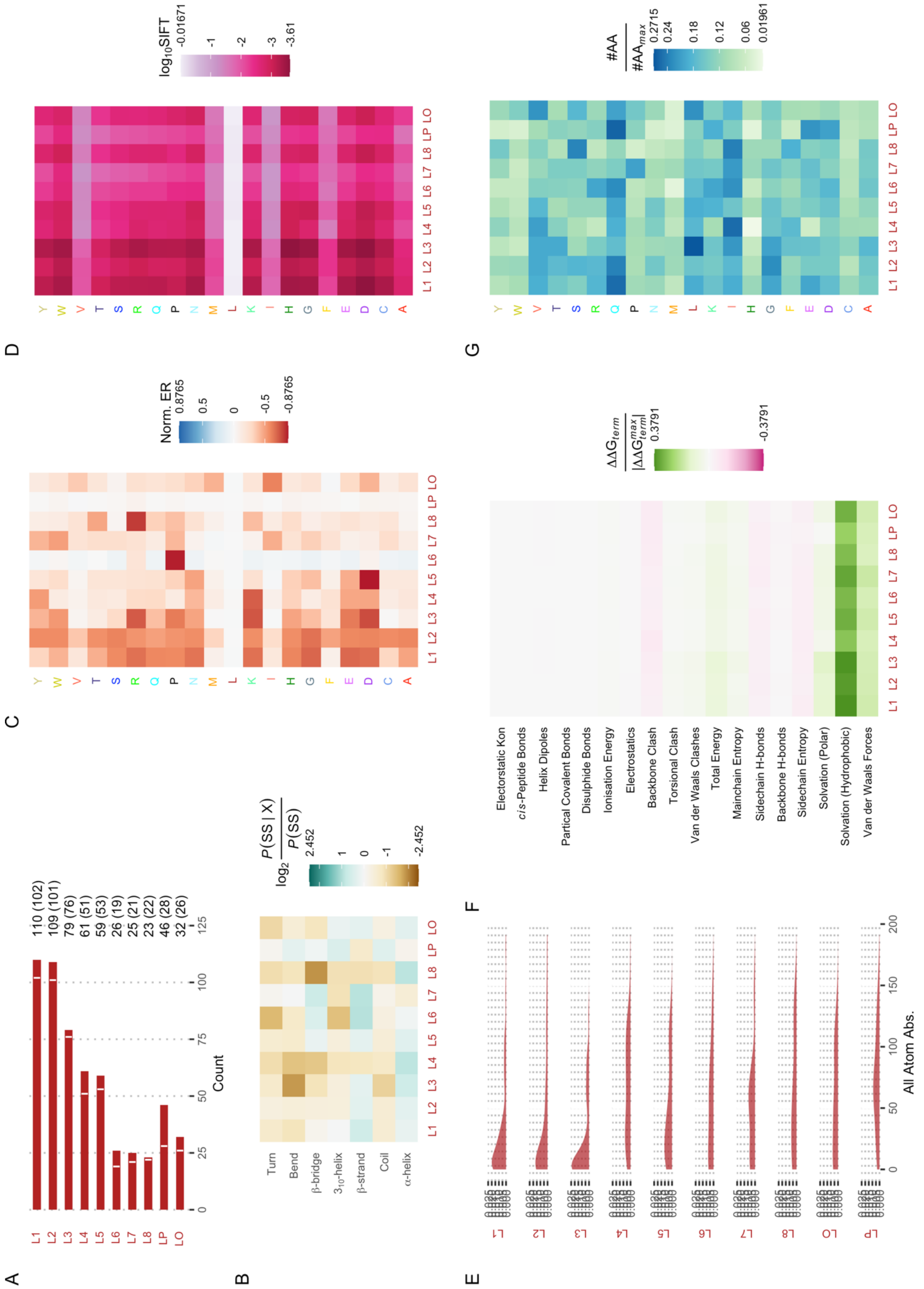

Figure S17 – Leucine Subtype Characterisation

Appendix Figure S18 – Methionine Subtype Characterisation

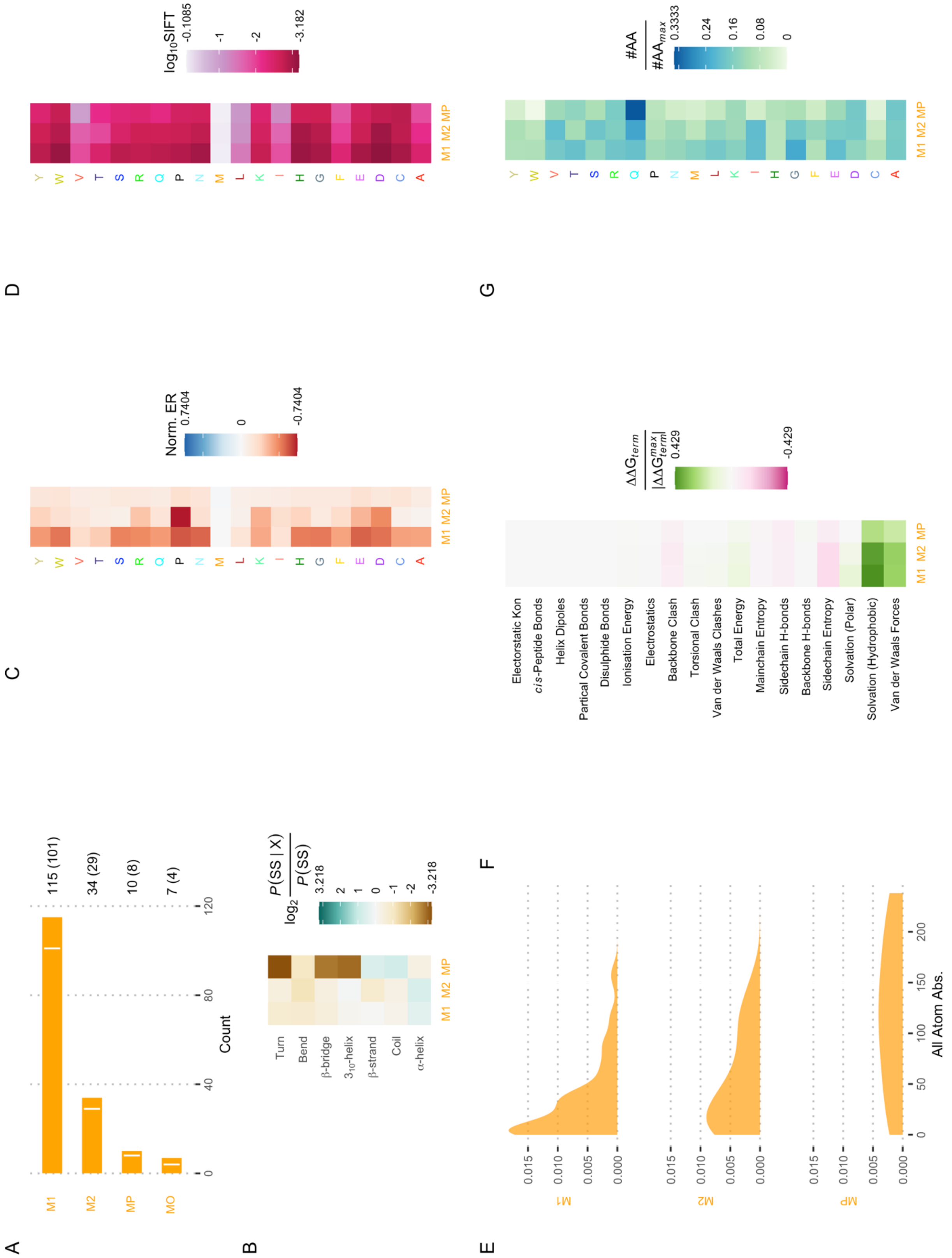

Figure S18 – Methionine Subtype Characterisation

Appendix Figure S19 – Asparagine Subtype Characterisation

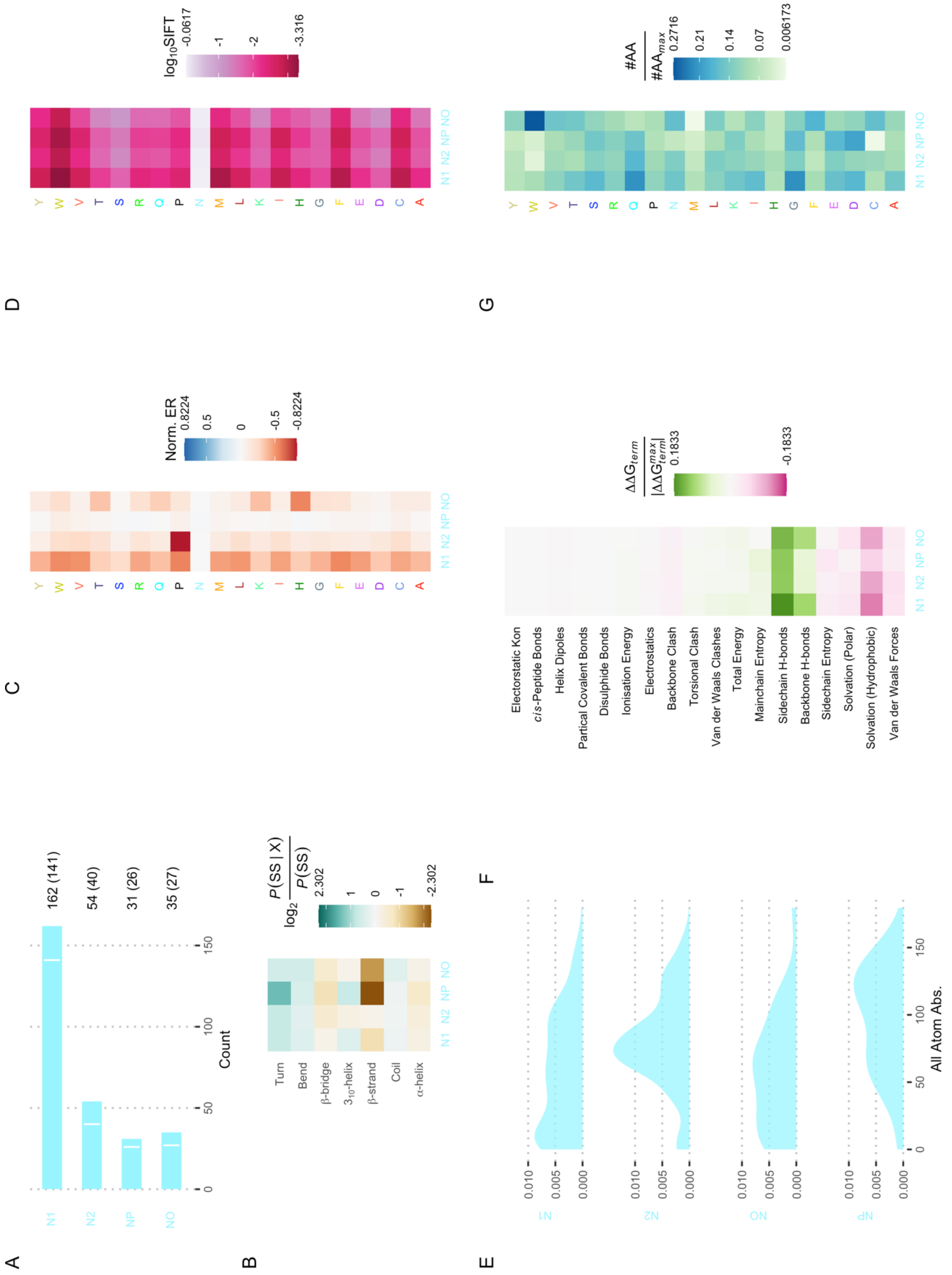

Figure S19 – Asparagine Subtype Characterisation

Appendix Figure S20 – Proline Subtype Characterisation

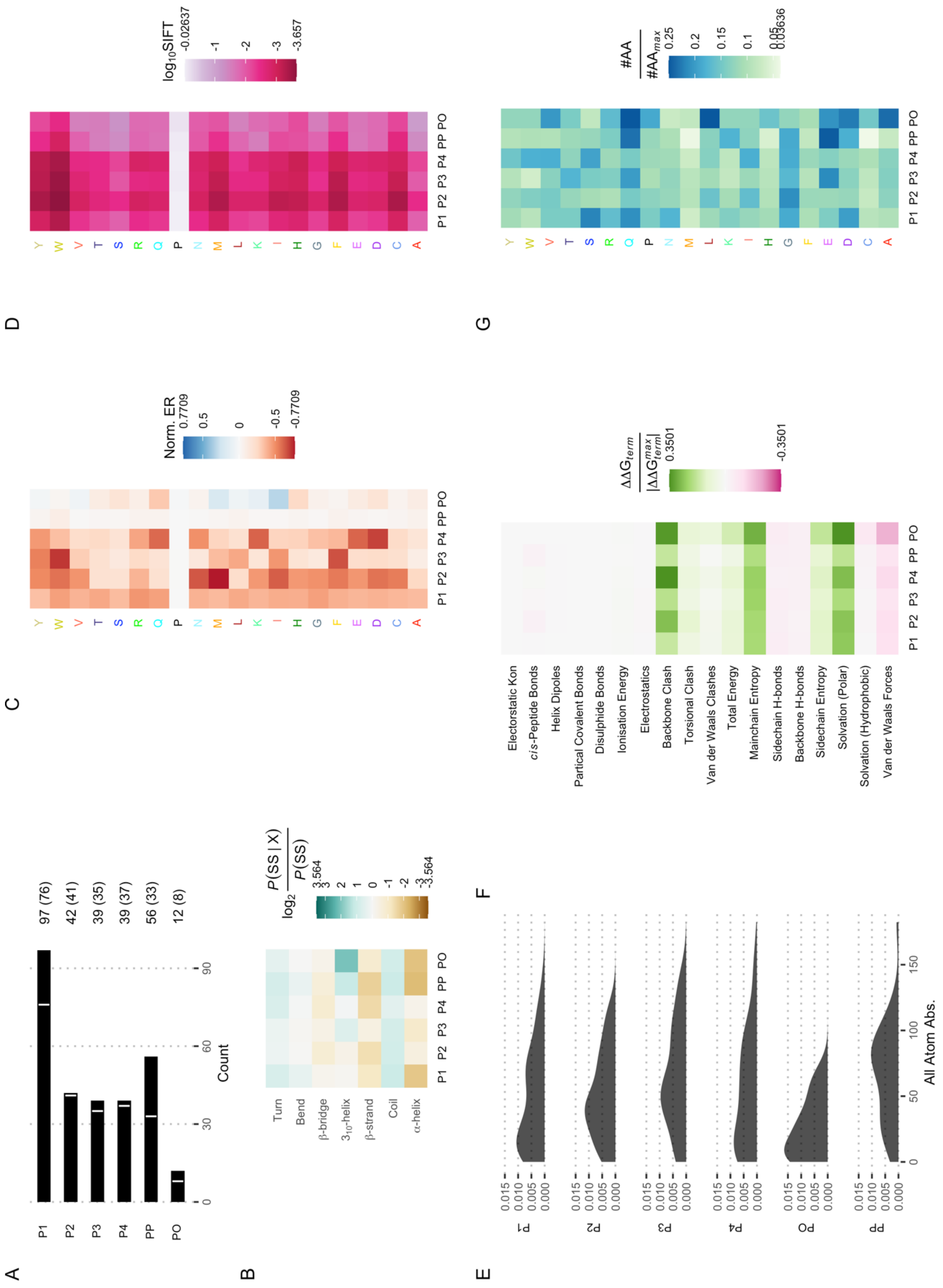

Figure S20 – Proline Subtype Characterisation

Appendix Figure S21 – Glutamine Subtype Characterisation

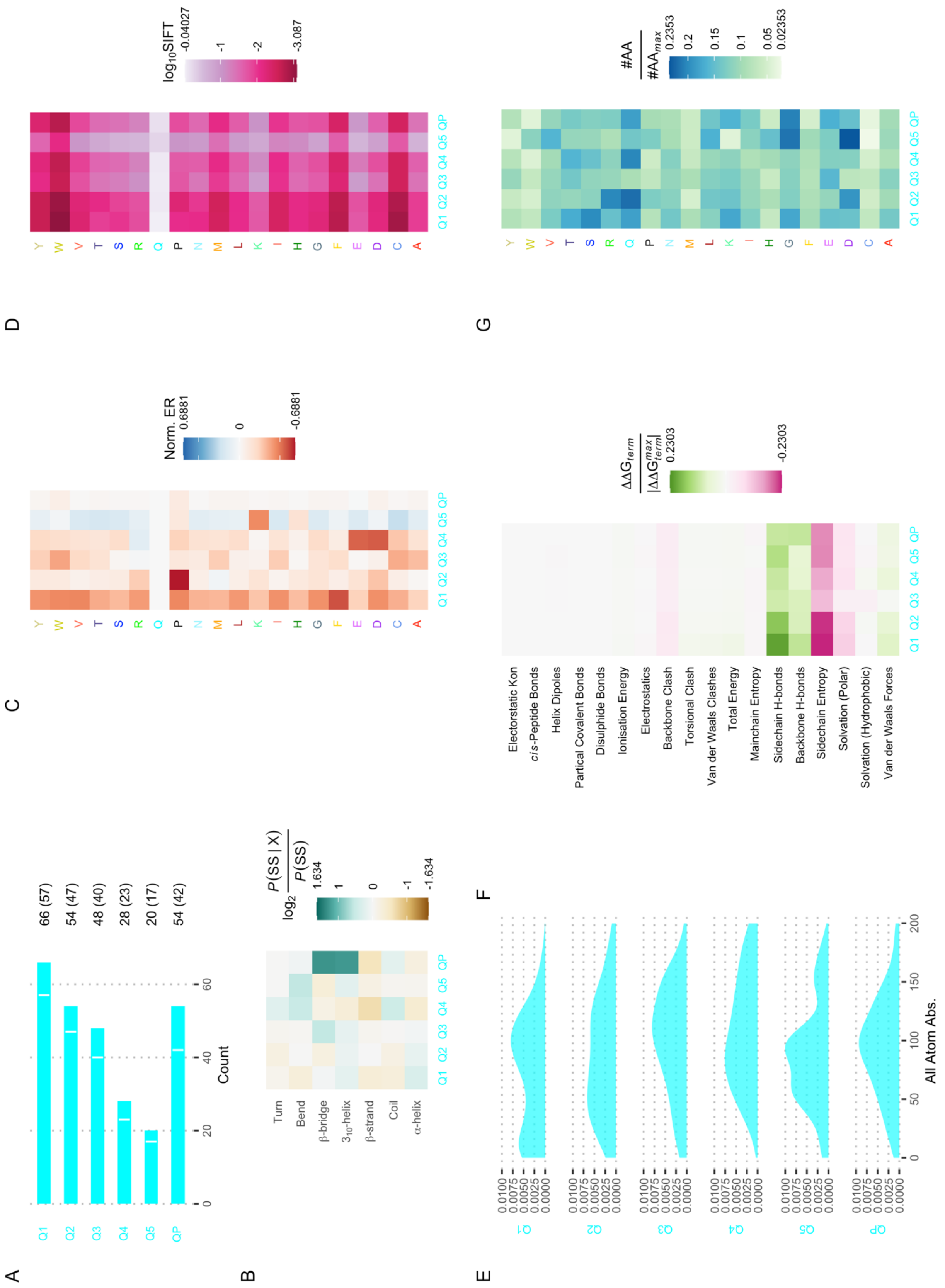

Figure S21 – Glutamine Subtype Characterisation

Appendix Figure S22 – Arginine Subtype Characterisation

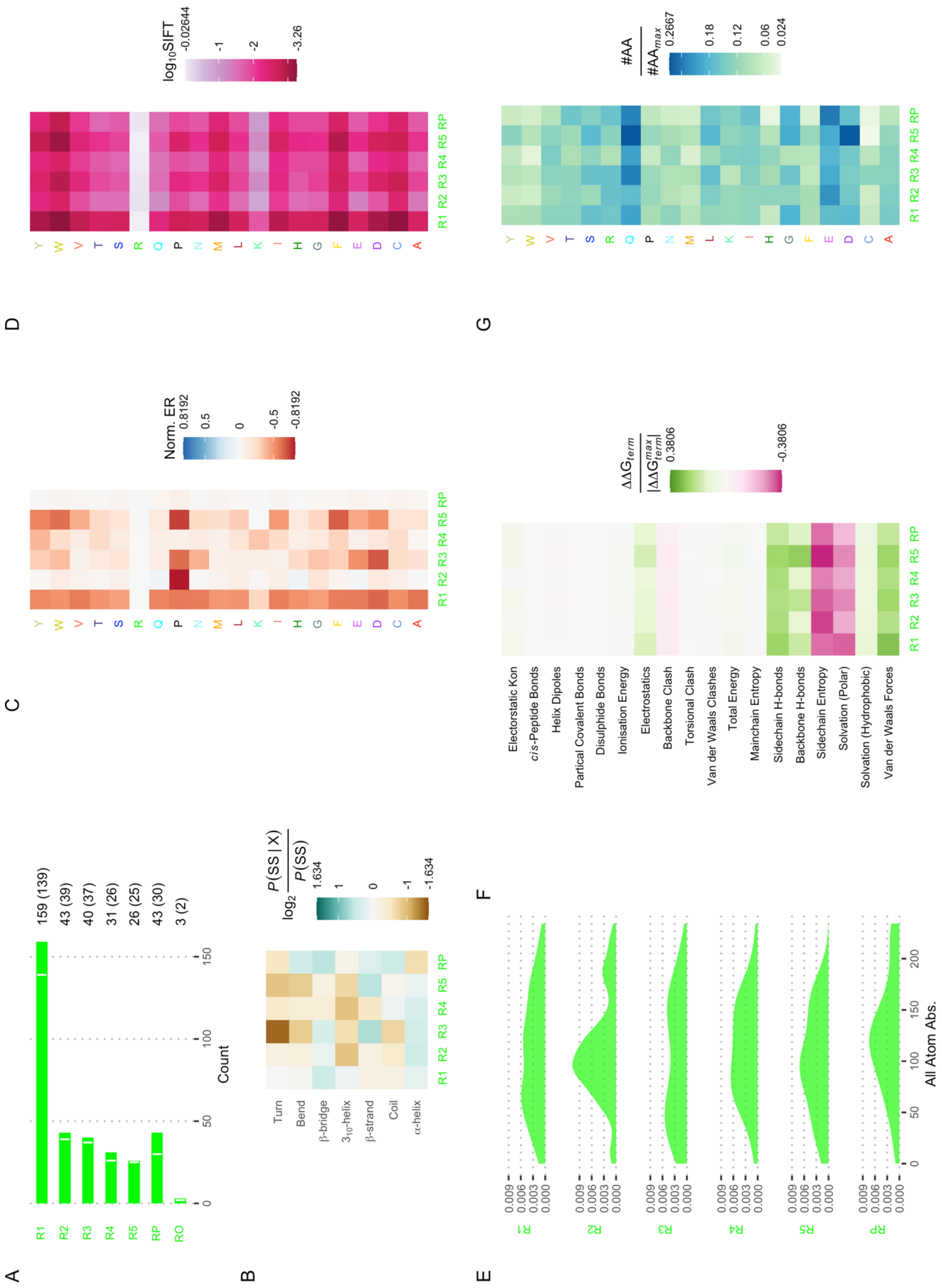

Figure S22 – Arginine Subtype Characterisation

Appendix Figure S23 – Serine Subtype Characterisation

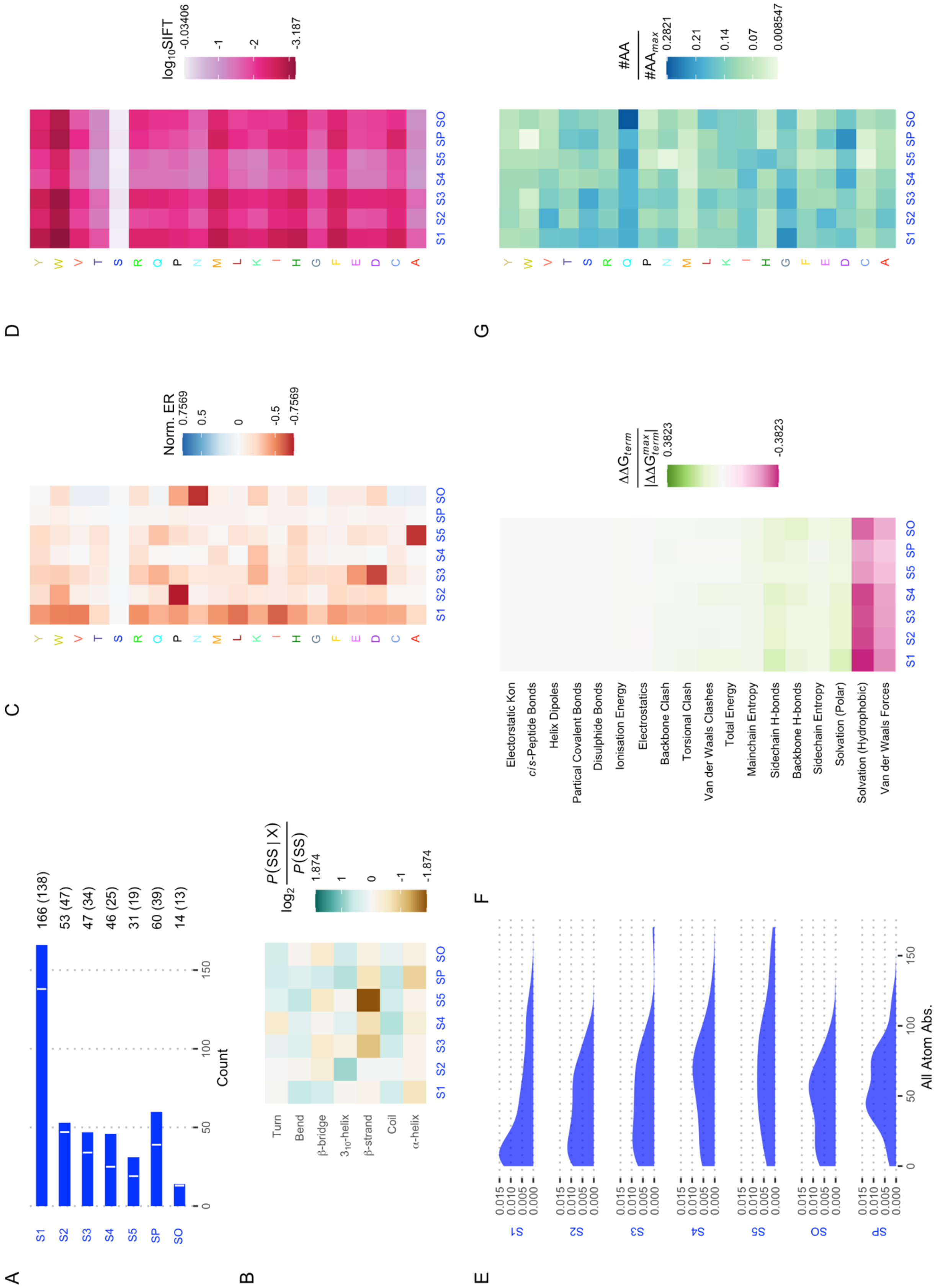

Figure S23 – Serine Subtype Characterisation

Appendix Figure S24 – Threonine Subtype Characterisation

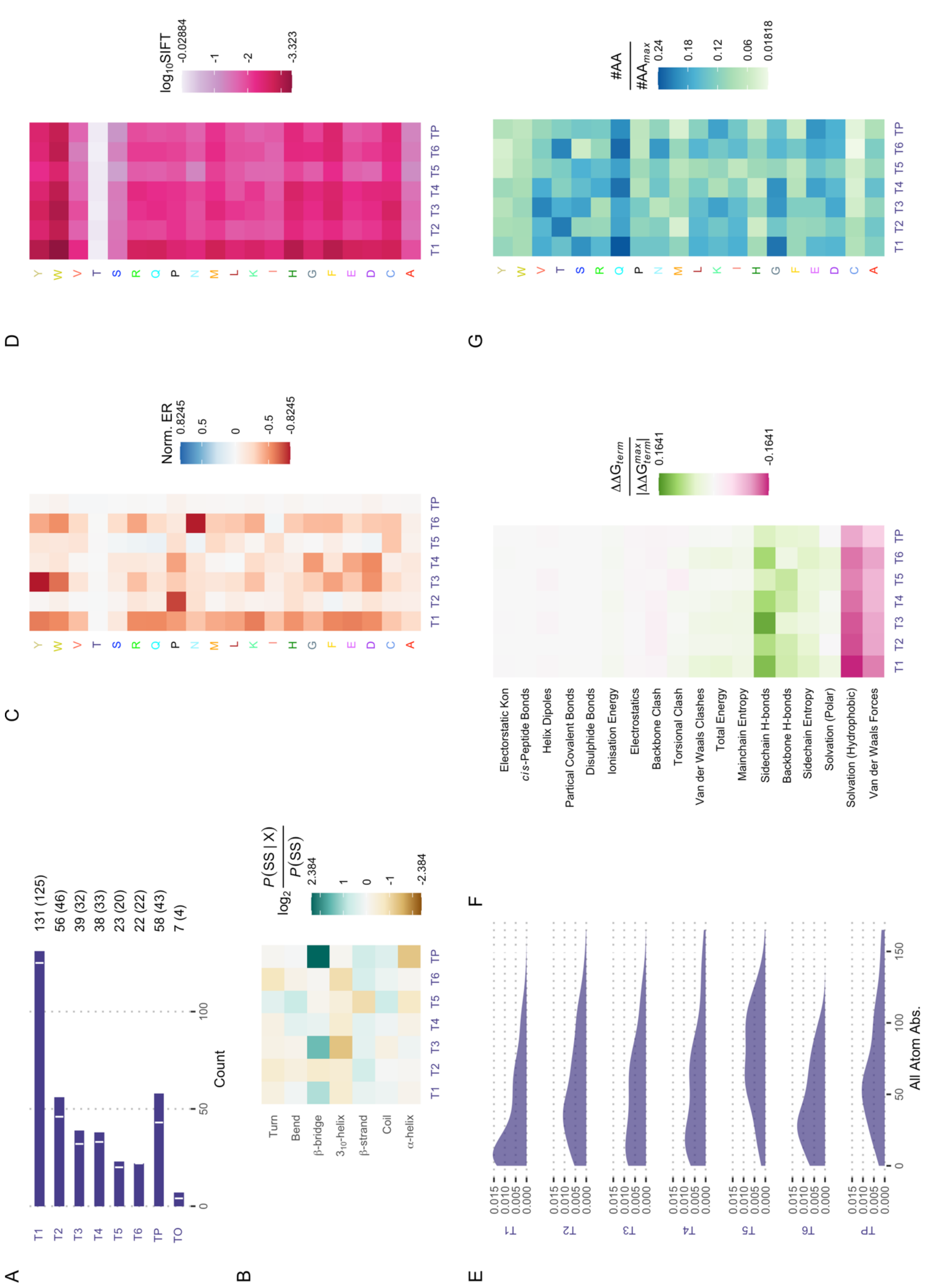

Figure S24 – Threonine Subtype Characterisation

Appendix Figure S25 – Valine Subtype Characterisation

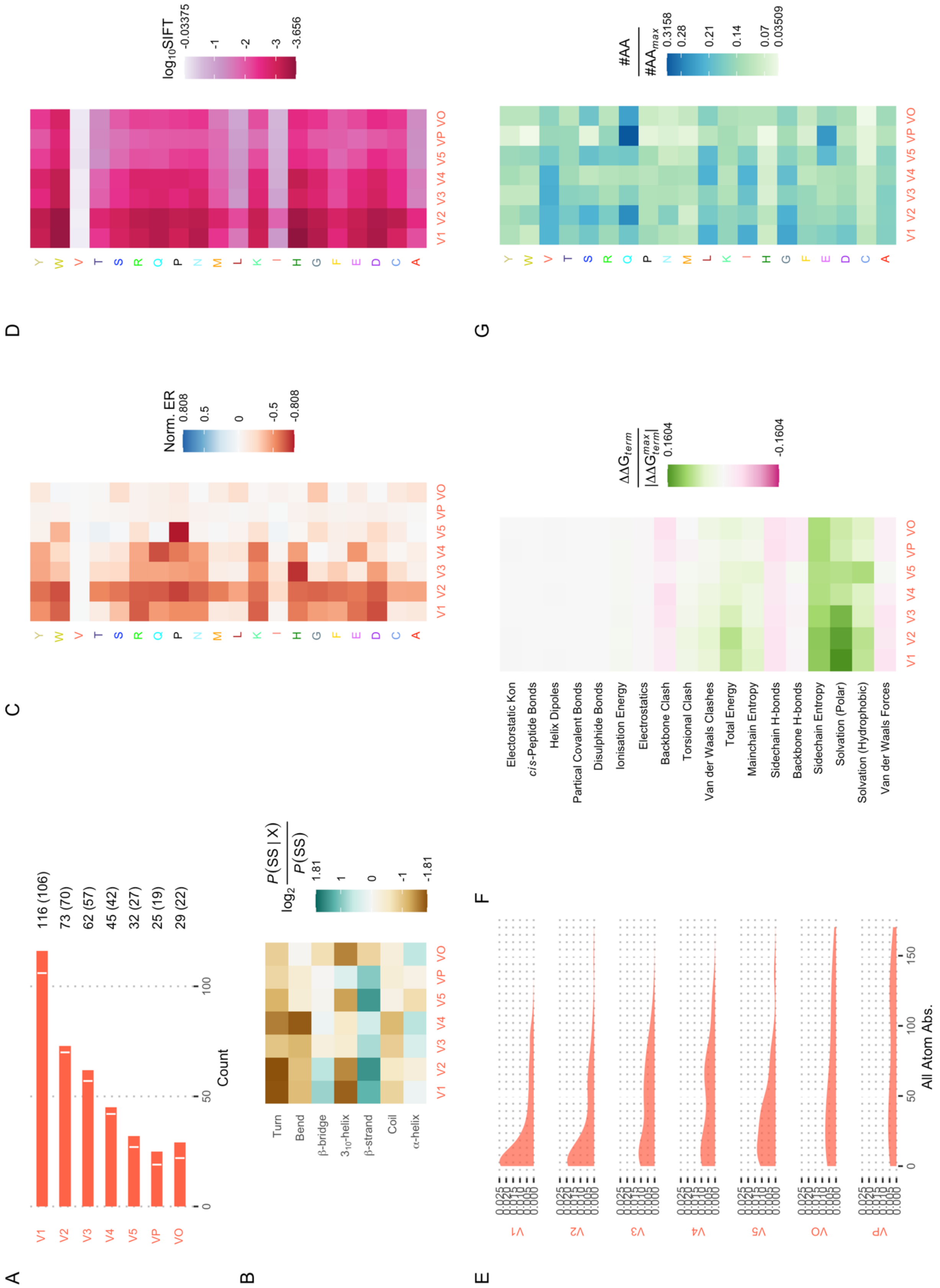

Figure S25 – Valine Subtype Characterisation

Appendix Figure S26 – Tryptophan Subtype Characterisation

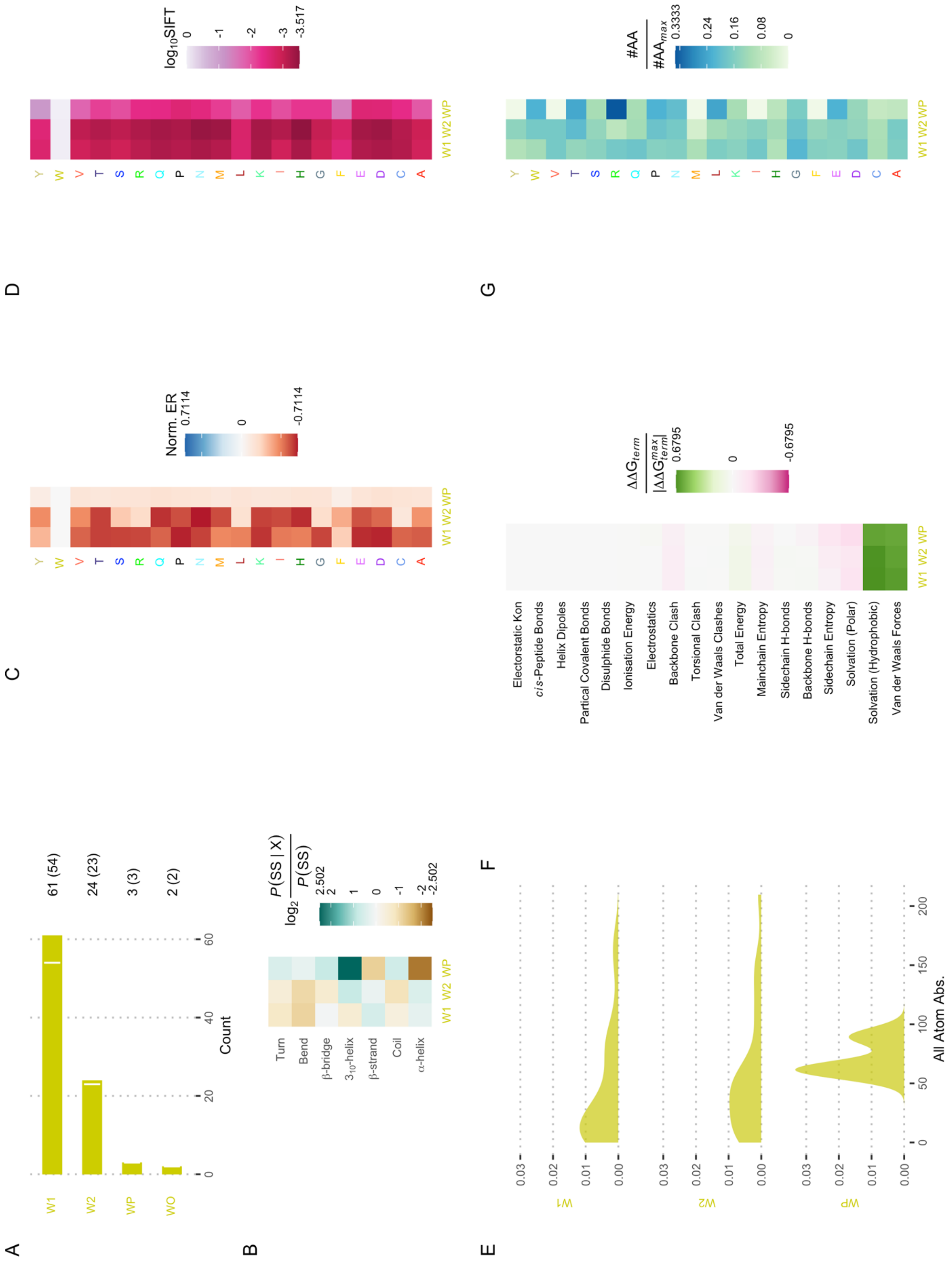

Figure S26 – Tryptophan Subtype Characterisation

Appendix Figure S27 – Tyrosine Subtype Characterisation

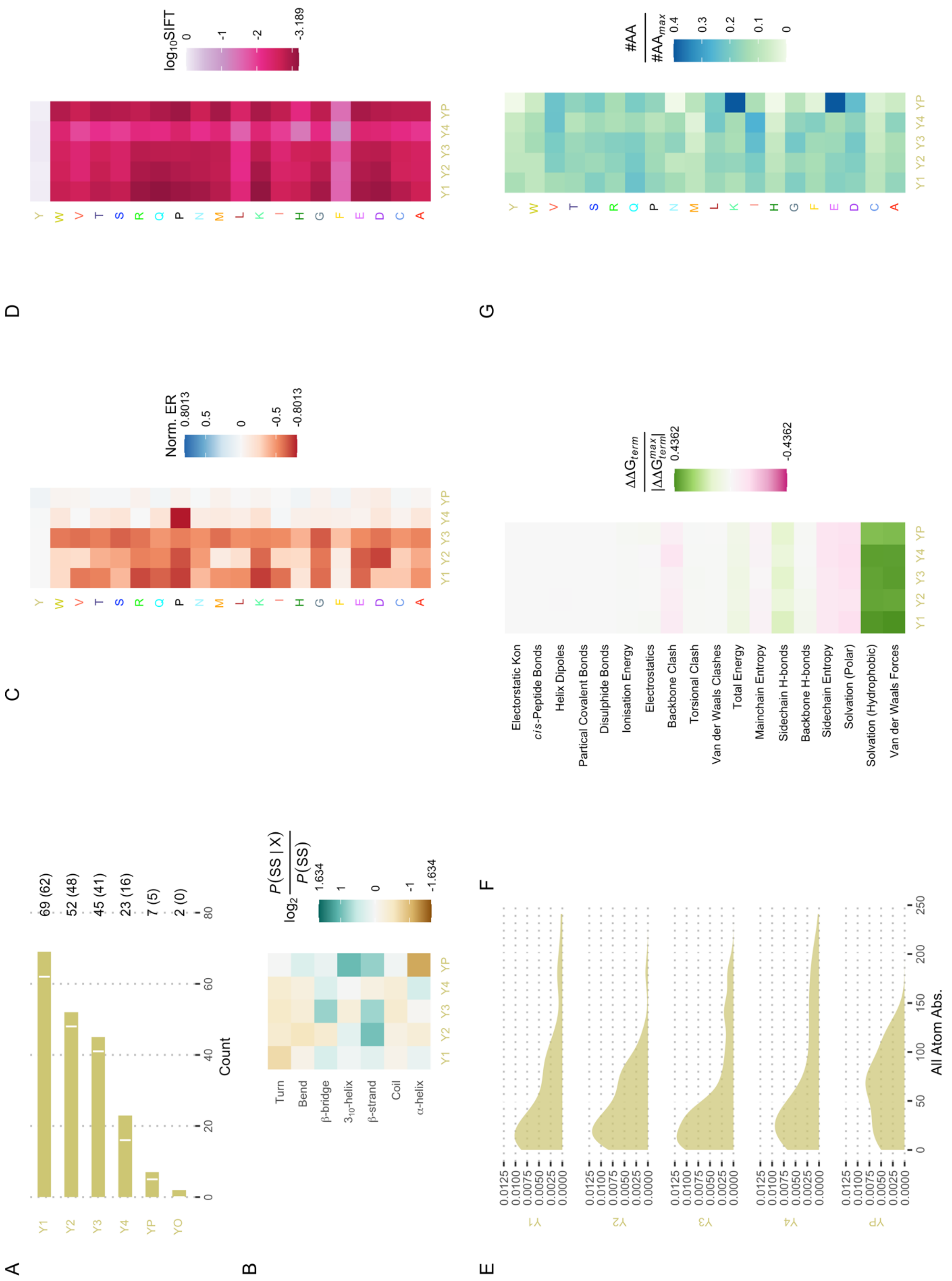

Figure S27 – Tyrosine Subtype Characterisation

Appendix Figure S28 – SIFT4G Mutational Landscape

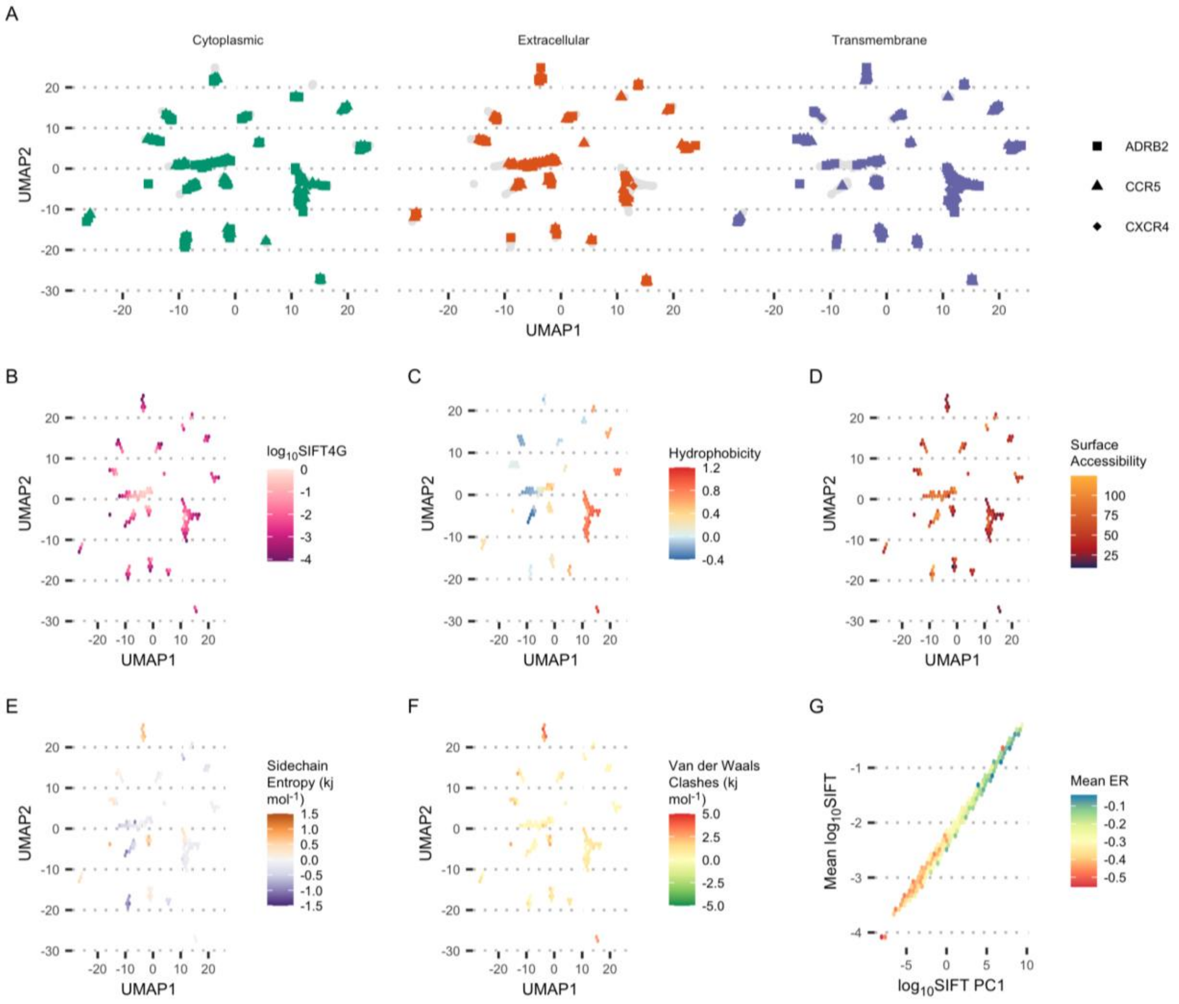

Appendix Figure S28 - Mutational landscape based on SIFT4G score profiles (as a proxy for evolutionary conservation). **A**-**F**: Features mapped to UMAP space calculated from SIFT4G score profiles. **A**: domains of ADRB2, CCR5 and CXCR4. **B**: mean log_{10}SIFT4G score. **C**: Average amino acid hydrophobicity. **D**: Surface accessibility **E**: Mean FoldX sidechain entropy $\Delta\Delta G$term for substitutions at each position **F**: Mean FoldX Van der Waals clashes $\Delta\Delta G$term for substitutions at each position. **G**: Correlation between PC1 (PCA on log_{10}SIFT4G score profiles) mean log_{10}SIFT4G score and Mean ER score.

Appendix Figure S29 – Comparison of cosine distance distribution for ER and SIFT4G based subtypes

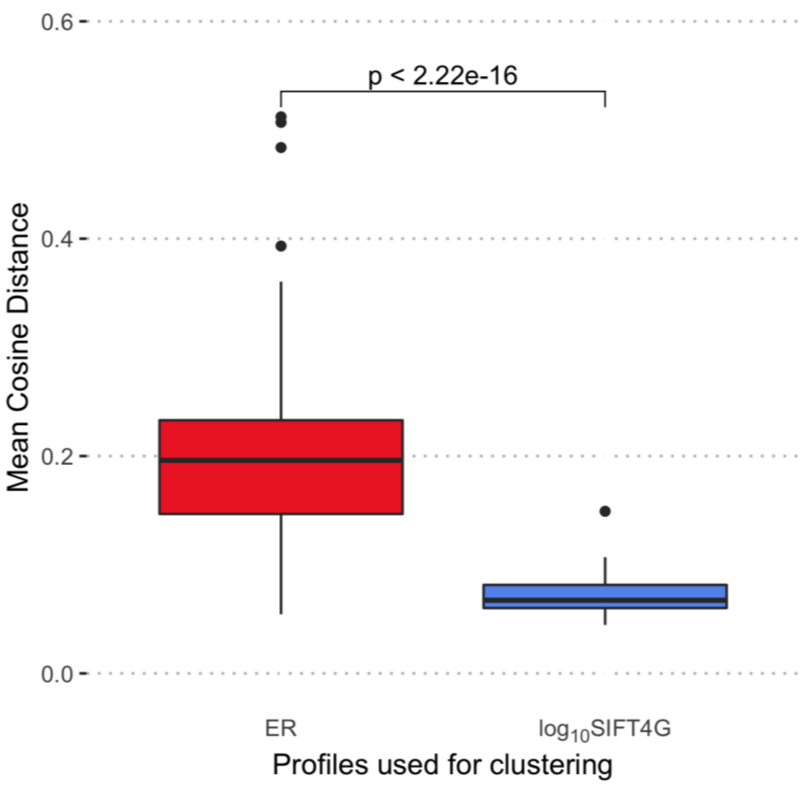

Appendix Figure S29 - Comparison of average cosine distance from each subtype's mean profile to other profiles of subtypes of the same amino acid. The profiles are ER scores for ER subtypes and log_{10}SIFT4G scores for SIFT4G based clusters. The statistical test is a one tailed Mann-Whitney U-Test with the alternative hypothesis that ER subtypes are on average more different to each other.
